# Validation of the reduced version of the Brazilian Scale of Moral Distress in Nurses

**DOI:** 10.1590/1518-8345.7123.4455

**Published:** 2025-02-17

**Authors:** Jordana Lopes Carvalho, Luis Felipe Dias Lopes, Flavia Regina Souza Ramos, Graziele de Lima Dalmolin

**Affiliations:** 1 Universidade Federal de Santa Maria, Santa Maria, RS, Brasil; 2 Universidade Federal de Santa Maria, Departamento de Ciências Administrativas, Santa Maria, RS, Brasil; 3 Universidade Federal de Santa Catarina, Florianópolis, SC, Brasil; 4 Universidade Federal de Santa Maria, Departamento de Enfermagem, Santa Maria, RS, Brasil

**Keywords:** Nursing, Nurses, Morale, Stress, Psychological, Metric System, Validation Study

## Abstract

**Objective:**

to validate a model of the reduced version of the Brazilian Scale of Moral Distress in Nurses.

**Method:**

methodological study, with a sample of 269 nurses from a public university hospital in southern Brazil. A sociodemographic questionnaire and the Brazilian Moral Distress Scale for Nurses were used. Descriptive statistics, structural equation modeling, and invariance analysis between age groups were used.

**Results:**

regarding profile, professionals aged between 31 and 40, women, married, and specialists prevailed. The structural model fitted the data with acceptable fit indices. The proposed model showed excellent internal consistency, convergent validity, and discriminant validity. The analysis of the structural model revealed that the five hypotheses were confirmed, and the invariance between the age groups indicates that the way the variables are conceptualized, measured, and interrelated in the model does not affect the results, regardless of the groups compared.

**Conclusion:**

the scale achieved satisfactory psychometric indicators, proving to be suitable for use, maintaining the relevant validation elements per dimension, and offering practical advantages. Further research is suggested to deepen our understanding of the consequences of moral distress and to develop effective strategies for managing it.

## Highlights

**(1)**The reduced version of the EDME-BR consisted of 24 questions and six factors. **(2)**Analysis of invariance and structural equation modeling were used for validation. **(3)**Internal consistency and composite reliability values were adequate. **(4)**The tests indicated sufficient predictive relevance of the proposed model. **(5)**The reduced scale offers a clear, accessible approach with a shorter response time.

## Introduction

In the context of professional nursing practice, nurses have to deal with everyday situations that require ethical and moral decisions, given the complexity of the health system. These decisions encompass organizational and structural aspects, as well as those related to teams and users, and demand positions and deliberations that arouse a range of emotions in professionals - such as restlessness, discomfort, and uncertainty in the face of conflicts. Such circumstances can culminate in the experience of Moral Distress (MD)^(1)^.

MD is conceptualized as the inner distress associated with external conflicts between values, obligations, and actions. It manifests itself when the nurse recognizes the appropriate line of conduct to follow but is prevented from acting according to their own discernment and judgment^(1)^.

Thus, the essential and sufficient conditions for defining MD are related to the reconciliation of moral experience and emotional disorder, in a direct causal relationship^(1)^. In the area of health, in the face of different ethically fragile circumstances, MD has stood out as a phenomenon or experience of great relevance to Nursing, due to its wide range of causes and meanings^(2-3)^.

Some contemporary international studies point to moderate levels of MD in hospital nurses^(3-4)^. These findings are in line with the results of a study conducted with Brazilian nurses working in the hospital environment, which reported a moderate level of frequency and intensity of MD^(5-6)^. However, as an aggravating factor, MD has implications that result in decreased job satisfaction, ethical challenges, increased workload and favor increased turnover of professionals^(7)^, which calls for a periodic evaluation of teams and work environments.

In this context, the progress of research requires continuous revisions and improvements in measurement instruments, which play a fundamental role in assessing moral distress among health professionals ^(8)^. MD is currently assessed using the Brazilian Moral Distress Scale for Nurses (MDSN-BR), which takes into account the peculiarities of the profession and the contexts in which services are provided in Brazil^(9)^.

This instrument showed the presence of MD with various predictive factors and pointed out the similarities between nurses’ experiences in the national and international context. It also addressed the care scenario at different levels of complexity and recognized the particularities of MD in Brazil^(9)^.

Considering the above, we reaffirm the importance of measuring the intensity and frequency of MD as an appropriate tool for the work environment and political-professional scenario, focusing on optimizing response time and facilitating evaluation in different contexts. This need is justified by the particularity of the MDSN-BR, as it is a scale made up of 49 items, which was used in a study in the research group with 269 nurses when the high response time and many refusals to participate were recognized because it was a long instrument. This led to the possibility, by using the study database, of carrying out a new methodological analysis to reduce the scale. The relevance of this is associated with the fact that greater knowledge about MD, using a scale that is easier to use, can contribute significantly to workers’ understanding of their circumstances and daily challenges.

With this in mind, the research question was: “Does the MDSN-BR have adequate validity and reliability in a reduced model?”. To answer this question, the aim was to validate a model of the reduced version of the MDSN-BR with professionals from a public hospital in southern Brazil.

The hypotheses for reducing the scale were the possible relationships between the scale’s dimensions, with subsequent contextualization:

H1: The perception of Recognition, Power, and Professional Identity (RPPI) is related to the ability to Defend Values and Rights (DVR);

H2: The recognition, power, and professional identity of nurses are related to the effectiveness and cohesion of Work Teams (WT);

H3: Working Conditions (WC) have a direct impact on the perception of WT;

H4: (Adverse) WC are related to the perception and occurrence of ethical infractions in the nursing environment;

H5: There is a relationship between the ability to provide Safe and Qualified Care (SQC) and the perception of Ethical Infractions (EIs);

H6: The analysis of invariance between the nurses’ age groups assumes that there is no difference between nurses aged up to 35 and nurses aged over 35 for the five hypotheses tested.

Finally, the hypotheses presented are in line with the literature, since: H1 suggests that nurses who feel more recognized and secure in their professional identity are more inclined to defend their values and rights in the workplace^(10)^; H2 suggests that nurses who perceive greater recognition of their competencies and professional value tend to contribute more effectively to teamwork, promoting a collaborative and mutually supportive environment ^(11)^; H3 suggests that better working conditions promote better team dynamics and cohesion ^(12)^; H4 suggests that work environments with insufficient resources, high workload, intense pressure, and inadequate organizational support increase the risk of ethical compromise, leading to decision-making that may not be in line with ethical and professional standards ^(13)^; and finally, H5 indicates that greater competence and safety in care are associated with a lower perception of ethical infractions in the work environment^(14)^. 

## Method

### Study design

This is a methodological study focusing on the validation of an instrument based on statistical modeling using the database of the project: “Moral distress in hospital nurses: what is its relationship with ethical climate and Burnout?” registered in the Project Portal of the University of origin of the researcher in charge. In the aforementioned analysis method, the quality of fit tests relates to how well a model fits the data. These tests help determine whether the model chosen reproduces the response variables for which the parameters have been optimized, or whether a different model needs to be considered^(15)^. 

### Scenario

The study was carried out at a public university hospital in southern Brazil. The institution currently has 400 beds and acts as a reference in the region for its technology and level of medium and high-complexity care, supported by a large team of workers and students in the area.

### Population, selection criteria, and sample definition

The participants were 269 nurses who took part in the aforementioned matrix project and made up its database. The matrix project had a population of 303 nurses working at the institution at the time of data collection, which was taken as the basis for using the formula to calculate the sample for the finite population, together with a sampling error of 5% and an estimated percentage of 50%; this resulted in a minimum of 171 participants. The inclusion criteria were nurses who had been working for at least one month (the minimum time estimated for perception of the work environment). Exclusion criteria were nurses who were on leave or away from work during the data collection period.

### Instruments used to collect information and study variables

The matrix project database consisted of a sociodemographic and work questionnaire, the MDSN-BR scale, the Maslach Burnout Inventory^(16)^, and the Hospital Ethical Climate Survey (HECS)^(17)^. However, for this study, we used the sociodemographic and work questionnaire variables and the MDSN-BR scale^(9)^. The socio-demographic and work questionnaire covered the following variables: gender, marital status, age (transformed into two age groups, i.e. up to 35 years and over 35 years), education, length of training, work shifts, and workload.

The MDSN-BR^(9)^ is an instrument built and validated in Brazil in 2019, which resulted in a final version of the scale with 49 items distributed in six factors, for which Cronbach’s alpha values ranged from 0.91 to 0.96^(9)^. These are: Factor 1 - RPPI, with 11 questions (14, 15, 16, 34, 35, 36, 37, 38, 40, 41, 43); Factor 2 - SQC, with 11 questions (23, 24, 25, 26, 27, 28, 29, 30, 31, 32, 33); Factor 3 - DVR, with eight questions (39, 42, 44, 45, 46, 47, 48, 49); Factor 4 - WC, with six questions (8, 9, 10, 11, 12, 13); Factor 5 - EIs, with six questions (17, 18, 19, 20, 21, 22); and Factor 6 - WT, with seven questions (1, 2, 3, 4, 5, 6, 7). A seven-point Likert scale was used for each item (from 0 = none/never to 6 = very intense/very frequent).

### Period and data collection

The database was accessed in May 2022. Data collection for the matrix project took place between April and June 2019. It was carried out by the authors and members of the research group who were not close to the participants, and who were previously trained and experienced in quantitative data collection.

The nurses were invited to take part in the research at their workplaces, choosing to answer the questionnaire immediately or hand it in later, after signing the Free and Informed Consent Term (FICT), which was signed by both the participant and the researcher, and a copy was given to each one.

All the nurses who agreed to take part in the study were given detailed information about the objectives and procedures, the potential risks and benefits involved, as well as being guaranteed the right to withdraw their consent to take part in the study at any time, without any negative repercussions or public exposure of their information.

If the professionals subsequently handed in the completed instruments, the search was scheduled and up to three attempts were made to retrieve the completed instruments, on different dates and at different times. In addition, during the completion process, the collectors ensured the freedom of the participants by only approaching them at the end to collect the instruments or to clarify any doubts.

To guarantee the confidentiality of the information, the completed forms were stored by the collectors in sealed envelopes and handed over to the researcher in charge.

### Data processing and analysis

The database was initially set up in the EpiInfo^®^ program (version 6.4), based on independent double entry, checking for errors and inconsistencies. The variables and scales selected from the database were transported for analysis in the SmartPLS^®^ (version 4.1.0.1) and Statistical Package for the Social Sciences (SPSS^®^ version 26) software. Sociodemographic and work-related variables were analyzed using descriptive statistics, with absolute (N) and relative (%) frequency distribution for categorical variables.

To validate the reduced scale, the structural equation modeling technique was used, using the partial least squares method, and Multigroup Analysis (MGA), more specifically, Invariance Analysis. The model chosen served the purpose of evaluating the structure of interrelationships between the dimensions of the proposed scale and one that had already been validated, by testing the proposed hypotheses^(18)^. In addition, the Partial Least Squares - Structural Equation Modeling (PLS-SEM) path model was used to analyze the data^(16)^.

This study used the Standardized Root Mean Square Residual (SRMR), the squared Euclidean distance (d_DEQ_), the geodesic distance (d_G_), and the Normed Fit Index (NFI) as criteria for assessing the fit of PLS-SEM. 0.08 was adopted as the threshold value for SRMR^(19)^and 0.8 as the minimum value for NFI^(20)^.

The MDSN-BR measurement model presented five direct hypotheses, which are connected in three dimensions (exogenous dimensions) with three endogenous dimensions (predictive) containing 24 items (observed variables) (Table 1). These hypotheses make it possible to assess the invariance of the reduced scale between age groups, affirming that the scale can measure what it is intended to.

In addition, Confirmatory Factor Analysis (CFA) comparing the age groups was carried out as an integral part of the PLS-SEM evaluation, to confirm and refine the items and dimensions laid out in the model. The internal consistency between the components was checked in each dimension using Cronbach’s alpha (CA). Discriminant validity was determined using the Fornell-Larcker (FL) and Heterotrait-Monotrait Ratio (HTMT) criteria, to determine the extent to which one dimension within its components differed from another^(21)^.

The square root of the Average Variance Extracted (AVE) value (Table 3) of each dimension was measured, compared with the Correlation Matrix values and the HTMT criterion, using the bootstrap procedure, which had values less than one for the upper limit (97.5% confidence).

Also at this stage, internal consistency reliability, convergent validity, and discriminant validity were checked^(20)^, i.e. the assumption of Average Variance Extracted (AVE > 0.5); Cronbach’s Alpha (0.7 < AC < 0.95) and composite reliability (0.7 < CC < 0.95) for the model^(22-23)^. To assess whether the structural model represented the theories underlying the measurement model, it was possible to analyze the model’s predictive capacity and the relationships between the dimensions of this model, and the (multi)collinearity analysis was considered by estimating the Variance Inflation Factor (VIF), which had values for the latent variables of less than 5^(22)^.

To assess the significance of the values of the path coefficients in the direct relationships between the factors, based on Student’s*t*-value, the bootstrapping technique was used with 5,000 subsamples. The direct relationship between the factors was measured using structural coefficients (β’s), and the path relationship was considered significant when a 5% significance level was obtained, with a*t*-value of less than 1.96^(18)^.

As for the reduction procedure, the techniques used to assess the structural model were used to check its internal consistency and convergent validity. When assessing the discriminant validity of the model, the most significant factor loadings (λ) for each of the dimensions were considered. The model was then submitted to an assessment of its structure, including analysis of accuracy (R^2^) and predictive relevance (Q^2^)^(18)^.

A crucial step in evaluating a PLS-SEM model is testing its accuracy and predictive relevance. Predictive accuracy was tested using the coefficient of determination (R2) and the respective p-value, which provides the degree of variance explained in each endogenous dimension^(18)^. The R2 value varies between 0 and 1 and the higher its value, the greater the predictive accuracy^(18)^. In addition, the significance of R2 was also assessed, with values indicating different levels of predictive accuracy: 0.075 < R2 ≤ 0.19 (moderate effect) and R2> 0.19 (strong effect)^(22-23)^.

Predictive relevance was assessed using the blindfolding procedure, which calculated the Q^2^value using the blindfolding technique. A Q^2^ value greater than 0 is considered indicative of predictive relevance^(18-23)^. Q^2^ values vary between 0.075 and 0.25 for a moderate degree of predictive relevance, while Q^2^ > 0.25 is indicative of a strong degree of predictive relevance^(23-24)^.

### Ethical aspects

All the ethical precepts of research involving human beings were respected, under Resolution No. 466/12, with the project approved by the local Research Ethics Committee (REC) under Opinion No. 2.764.702.

## Results

### Participants’ profile

A total of 269 nurses took part in the survey, of whom 83 (30.9%) were up to 35 years old and 186 (69.1%) were over 35. The majority of participants were female (88.1%), and 75.09% were married. As for academic training, 54.37% of the participants had a specialization degree and 53.17% had completed their training between six and 15 years before the survey. Concerning working hours, the majority of nurses (65.06%) worked 30 hours or more a week, and 39.04% had mixed shifts. It is worth noting that, when considering that time working at the hospital was an inclusion criterion, the median time working at the institution was four (AIq10) years, 187 (69.5%) had four years or more, and only four (1.49%) had worked for one month, while all the others had worked for over six months.

### Model fit tests

The results confirmed that the suggested structural model fitted the data after eight interactions with acceptable indices, with SRMR = 0.062, d_DEQ_ = 2.563, d_G_ = 1.605, and NFI = 0.848.

### Assessment of the measurement model

At this stage, the three criteria were assessed: internal consistency reliability, convergent validity, and discriminant validity, including the average variance extracted.

The internal consistency between the components in each dimension was checked using the CA and CR. The CA values for all the dimensions ranged from 0.798 to 0.930. In addition, the Composite Reliability (CR) values ranged from 0.817 to 0.947. Finally, the AVE values ranged from 0.701 to 0.950. As for the factor loadings, for the MDSN-BR-R scale, the factor loadings were higher than 0.6 (Figure 2), so these results confirmed the guarantee of internal consistency between the indicators and their respective factors. Table 1 shows the evaluation values for all the dimensions of the measurement model.

When assessing the model for discriminant validity, the main criteria used were FL and HTMT. The FL criterion compared the square root of the AVE value of each dimension measured to the values of the correlation matrix. The lowest AVE (0.802) was observed to be higher than the highest WC vs. SQC correlation (0.679), so the criterion was established. The HTMT criterion determined that the estimated upper limits were less than 1.0. The model was therefore found to have discriminant validity and thus met the requirements.

In summary, the evaluations of the measurement model in terms of internal consistency, convergent validity, and discriminant validity met their requirements, empirically validating the suitability of the measurement model in this study (Tables 1 and 2).

**Table 1 -:** Assessment of the measurement model - internal consistency and convergent validity. Santa Maria, RS, Brazil, 2019

MDSN-BR* (Dimensions)	**CA** ^†^		**CR** ^‡^		**AVE** ^§^	
	**≤ 35** ^ǁ^	**> 35** ^¶^	**≤ 35** ^ǁ^	**> 35** ^¶^	**≤ 35** ^ǁ^	**> 35** ^¶^
RPPI**	0.857	0.798	0.904	0.817	0.701	0.865
DVR^††^	0.875	0.883	0.912	0.904	0.721	0.919
EIs^§§^	0.898	0.930	0.929	0.936	0.766	0.950
SQC^‖‖‖^	0.925	0.899	0.947	0.900	0.816	0.930
WCs ^¶¶^	0.868	0.825	0.909	0.835	0.714	0.882
WTs**	0.785	0.830	0.861	0.838	0.608	0.887

*MDSN-BR = Brazilian Moral Distress Scale for Nurses;^†^CA = Cronbach’s Alpha;^‡^CR = Composite Reliability;^§^AVE = Average Variance Extracted;^ǁ^≤ 35 = Less than and equal to 35 years;^¶^> 35 = Greater than 35 years; **RPPI = Recognition, Power and Professional Identity;^††^DVR = Defense of Values and Rights;^§§^EIs = Ethical Infractions;^ǁǁ^SQC = Safe and Qualified Care;^¶¶^WCs = Working Conditions; ***WTs = Work Teams

**Table 2 -:** Fornell-Larcker criterion and HTMT* of the factorial model. Santa Maria, RS, Brazil, 2019

**Dim.** ^†^	^‡^	**Pearson Correlation Matrix**					
		**RPPI** ^§^	**DVR** ^‖‖^	**EIs** ^¶^	**SQC** ^**^	**WCs** ^††^	**WTs** ^‡‡^
RPIP^§^	0.821	1.000					
DVR^‖‖^	0.885	0.632	1.000				
EIs^¶^	0.856	0.476	0.607	1.000			
SQC**	0.805	0.586	0.527	0.561	1.000		
WCs^††^	0.897	0.555	0.664	0.674	0.679	1.000	
WTs^‡‡^	0.802	0.551	0.576	0.575	0.665	0.647	1.000
		Upper limit (HTMT*)_97.5%_					
DVR^‖‖^		0.733					
EIs^¶^		0.811	0.816				
SQC**		0.742	0.758	0.790			
WCs^††^		0.740	0.648	0.707	0.821		
WTs^‡‡^		0.866	0.738	0.873	0.709	0.785	

*HTMT = Heterotrait-Monotrait Ratio;^†^Dim. = Dimension;^‡^ = Root Mean Variance Extracted;^§^RPPI = Recognition, Power and Professional Identity;^‖‖^DVR = Defense of Values and Rights;^¶^EIs = Ethical Infractions; **SQC = Safe and Qualified Care;^††^WCs = Working Conditions;^‡‡^WTs = Work Teams

### Analysis of the structural model

After analyzing the collinearity between the dimensions and the VIF values were calculated between 1.000 < VIF < 1.664, the model did not show strong correlations, so the values of the regression coefficients (β’s) were estimated using the bootstrapping technique for 5,000 subsamples. The values and their significance are shown in Table 3.

**Table 3 -:** Results of direct effects between dimensions. Santa Maria, RS, Brazil, 2019

**Path relationship (Hypotheses)**		**Path coefficients**	**Standard Deviation**	Stat. t.*	**p-value** ^†^	**Result**
H1^‡^	RPPI^§^→ DVR^‖‖^	0575	0.040	14.526	0.000	Supports
H2^¶^	RPPI^§^→ WTs**	0.492	0.056	8.763	0.000	Supports
H3^††^	WCs^‡‡^→ WT**	0.315	0.059	5.288	0.000	Supports
H4^§§^	WC^‡‡^→ EIs^‖‖‖‖^	0.226	0.062	3.652	0.000	Supports
H5^¶¶^	SQC*** → WC^‡‡^	-0.521	0.056	9.349	0.000	Supports

*Stat. T = Student’s t-test;^†^p-value = Significance;^‡^H1 = Hypothesis 1;^§^RPPI = Recognition, Power and Professional Identity;^ǁ^DVR = Defense of Values and Rights;^¶^H2 = Hypothesis 2;^**^WTs = Work Teams;^††^H3 = Hypothesis 3;^‡‡^WC = Working Conditions;^§§^H4 = Hypothesis 4;^ǁǁ^EIs = Ethical Infractions;^¶¶^H5 = Hypothesis 5;^***^SQC = Safe and Qualified Care

**Figure 1 -:**
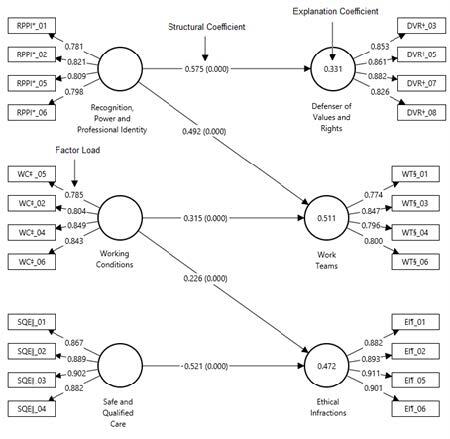
Final structural equation model. Santa Maria, RS, Brazil, 2019

All the proposed hypotheses confirmed significant relationships (p < 0.05) between the dimensions, so the hypotheses were empirically supported based on the*t*-values for a 5% significance level. It was observed that safe and qualified care is negatively related to ethical infractions; ten relationships were significant (Figure 1).

The analysis (Figure 1) revealed that the model showed dimensions with coefficients of determination (R^2^) indicating strong degrees of explanation (R^2^ > 0.19) for the predictive variables, which affirms that these results were consistent. In addition, the predictive relevance values of the endogenous dimensions, estimated using the blindfolding method, showed Q^2^ values greater than 0, indicating a good predictive capacity of the model. More specifically, the Q^2^ values ranged from 0.323 to 0.498.

Table 4 below shows the model’s invariance analysis for Hypothesis H6, which compares hypotheses H1 to H5 between the age groups up to 35, compared to the age group over 35.

*RPPI = Recognition, Power and Professional Identity;^†^DVR = Defense of Values and Rights;^‡^WCs = Working Conditions;^§^WTs = Work Teams;^ǁ^SQC = Safe and Qualified Care;^¶^EIs = Ethical Infractions

**Table 4 -:** Multi-group analysis (MGA*) by Henseler’s and Permutations tests. Santa Maria, (RS), Brazil. 2019

**Hip.** ^‡^	**Relationship**	**Structural Coefficients**			**p-value** ^†^ **(diferences)**		**Result**
		**Ut.35** ^§^	**Ov.35** ^ǁ^	**Ut-Ov** ^¶^	Henseler’s MGA**	**Permutation test**	
H1^††^	RPPI^‡‡^→ DVR^§§^	0.640	0.549	0.090	0.283	0.306	No / No
H2^ǁǁ^	RPPI^‡‡^ → WTs^¶¶^	0.426	0.516	-0.091	0.429	0.469	No / No
H3***	WCs^†††^ → WTs^¶¶^	0.371	0.312	0.059	0.625	0.642	No / No
H4^‡‡‡^	WCs^†††^ → EIs^§§§^	0.189	0.245	-0.057	0.679	0.688	No / No
H5^ǁǁǁ^	SQC^¶¶¶^ → WCs^†††^	-0.620	-0.477	-0.143	0.245	0.222	No / No

*MGA = Multi Group Analysis;^†^p-value = Significance; ‡Hypotheses;^§^Ut. 35 = Up to 35 years old;^ǁ^Ov. 35 = Over 35 years old;^¶^Ut - Ac = Difference between up to and over 35 years old;^††^H1 = Hypothesis 1;^‡‡^RPPI = Recognition, Power and Professional Identity;^§§^DVR = Defense of Values and Rights;^ǁǁ^H2 = Hypothesis 2;^¶¶^WTs = Work Teams; ***H3 = Hypothesis 3;^†††^WCs = Working Conditions;^‡‡‡^H4 = Hypothesis 4;^§§§^EIs = Ethical Infractions;^ǁǁǁ^H5 = Hypothesis 5;^¶¶¶^SQC = Safe and Qualified Care

Table 4shows that, for both the Henseler tests and the permutations test, there is no significant difference between the coefficients of the different age groups (p > 0.05). Therefore, it can be concluded that the proposed model does not present problems of invariance and thus the scale behaves consistently between the groups^(25)^.

Cronbach’s alpha for the MDSN-BR model showed good reliability in all its factors (0.95), which is lower than the original version (0.98). The reduced version of the MDSN-BR, containing 24 items, was obtained by calculating the reliability indices (CA and CR), considering the four items per dimension with the highest factor loadings as the criteria for selecting the indicators.

In addition, to preserve the scale’s characteristics, the changes made did not modify the instructions and response scale or interfere with the quality of the results obtained. Therefore, the reduced version of the MDSN-BR was made up of 24 questions assessing the following factors:

Factor 1 - Recognition, Power, and Professional Identity (RPPI), with four questions: 14) Feeling discriminated against concerning other professionals; 36) Working under pressure due to insufficient time to achieve goals or carry out tasks; 40) Feeling disrespected by hierarchical superiors; 41) Recognizing ethically incorrect attitudes of managers or hierarchical superiors.

Factor 2 - Safe and Qualified Care (SQC), with four questions: 24) Recognize that user reception is inadequate; 25) Recognize that patient/user demands for continuity of care are not met; 26) Recognize the lack of resolution of health actions due to social problems; and 28) Recognize that educational actions with the user are insufficient.

Factor 3 - Defense of Values and Rights (DVR), with four questions: 39) Experiencing care conduct that disregards patients’ beliefs and culture; 42) Feeling pressured to agree to or remain silent in the face of fraud for the benefit of the institution; 45) Recognizing situations of disrespect/mistreatment by professionals towards the user; and 46) Recognizing situations of disrespect for the user’s right to privacy/intimacy.

Factor 4 - Working conditions (WCs), with four questions: 8) Recognize that consumables are insufficient; 10) Recognize that the permanent equipment/materials available are insufficient; 12) Recognize that the physical structure of the service is insufficient; and 13) Recognize that the physical structure of the service is inadequate.

Factor 5 - Ethical Infractions (EIs), with four questions: 17) Experiencing omission on the part of the doctor; 18) Experiencing recklessness on the part of the doctor; 19) Experiencing omission on the part of the nurse; and 20) Experiencing recklessness on the part of the nurse.

Factor 6 - Work Teams (WTs), with four questions: 1) Working with an insufficient number of professionals for the demand; 3) Experiencing conditions of work overload; 5) Working with unprepared nurses; and 6) Working with unprepared nursing assistants and technicians.

A seven-point Likert scale was used for each item (from 0 = none/never to 6 = very intense/very frequent).

## Discussion

Based on the results, it was possible to validate the MDSN-BR in its reduced version, which was called the “Brazilian Scale of Moral Distress in Nurses - Reduced Version” (MDSN-BR-VR), which presented a reduction of 25 questions compared to the original version. The MDSN-BR-VR remained with 24 questions, keeping the factors: Recognition, Power, and Professional Identity; Safe and Qualified Care; Defense of Values and Rights; Working Conditions; Ethical Infractions; and Work Teams. However, each one now consists of just four items.

In this model, the study obtained SRMR = 0.062 and NFI = 0.848. The root mean square of the standardized residuals is a measure of fit that aims to assess the degree of adequacy of the model and must be less than 0.08 for a satisfactory result^(19- 20)^. The NFI is the ratio between the χ² of the independent model and the χ² of the model that was tested, does not penalize the complexity of the model, and is sensitive to the size of the sample. It is a comparison between the covariance matrix predicted by the CFA and the original matrix of what is predicted by the model and must be greater than 0.8^(19-20)^.

As for the hypotheses presented and found, the relationship between RPPI and DVR can be explained by the concept of professional empowerment, which involves having the power, authority, and capacity to carry out actions based on ethical and professional values. Nurses who perceive greater empowerment at work feel more empowered to act under their professional values, which includes defending patients’ rights and adhering to high ethical standards^(26- 27)^.

A strong professional identity and recognition in the workplace contribute to better team dynamics. Clarity of role and recognition of nurses’ skills are associated with better communication and collaboration between team members, facilitating group cohesion and effectiveness^(28)^.

The influence of working conditions on the perception of nursing teams can be understood through the theory of work resources. This theory postulates that work environments with functional resources improve employee satisfaction and engagement and promote positive relationships between colleagues. Better working conditions are associated with better quality of care and job satisfaction among nurses^(29)^.

It is known that adverse working conditions can create ethical dilemmas, where nurses are forced to make choices that compromise ethical standards. Stressful environments with insufficient resources increase the ethical conflicts faced by nurses, highlighting the relationship between working conditions and the occurrence of ethical infractions^(30)^.

The ability to provide safe and qualified care can be compromised in environments where ethical violations prevail, creating a negative relationship between these dimensions. In other words, the impacts resulting from MD can compromise nurses’ performance and negatively influence their effectiveness in meeting established health objectives^(13)^.

The fact that all these hypotheses did not differ between the age groups up to 35 and over 35 suggests a universality in nurses’ perceptions and experiences, regardless of age. This may indicate that professional, ethical, and organizational structures are perceived consistently throughout the nursing career, reinforcing the importance of addressing these dimensions in professional development strategies and health policies.

An international study of nursing professionals showed significant levels of intensity and frequency of moral distress due to inadequate working conditions^(31)^.

The hypotheses of professional fulfillment and MD did not imply their scores. This corroborated the findings of a study carried out with 280 nurses, which looked at the relationship between job satisfaction and moral distress. Satisfaction is an inverse construct to MD, i.e. the higher the degree of MD, the lower the professional fulfillment^(32)^.

Moral distress and job satisfaction scores showed a negative correlation between their variables, indicating that these constructs are inversely proportional^(33)^. Levels of professional chievement can be associated with organizational aspects, interactions, autonomy, and remuneration, which, at low levels, can lead to physical manifestations, due to work overload, or emotional manifestations, such as stress and anxiety, which often generate job dissatisfaction and moral distress^(7)^.

Feelings of devaluation and powerlessness on the part of professionals can lead to ethical/moral conflicts, leading to job dissatisfaction^(33)^. However, it was possible to see in the reduced ersion of the scale that the highest MD averages were linked to the constructs of safe and qualified care, and working conditions, followed by ethical infractions.

These results are in line with other studies carried out using the original scale. A study of hospital nurses showed that the highest levels of moral distress were related to the constructs “safe and qualified care”, “working conditions” and “work teams”^(34)^.

In addition, of the 269 nurses who took part, the majority were female and the prevalence was between 31 and 40 years old. A study that found MD in nurses obtained similar results, with a profile represented mostly by women, with an average age of 37^(35)^.

The occurrence of MD is associated with the subjective nature of work, professional experience, and organizational conditions, especially with the challenges faced in daily work^(8)^. An event that riggers MD, whether frequent or sporadic, can provide important data that should be analyzed, with emphasis on the relevance of the circumstances surrounding it.

Although various definitions of MD have been suggested in the literature, there is agreement that it emerges from inadequate limitations in clinical practice that impact the moral autonomy of health professionals. Thus, to mitigate moral distress, measurement instruments must provide relevant information about these limitations^(36)^, with the MDSN-BR-VR being a valid and reliable option.

When comparing the results obtained from the MDSN-BR and the MDSN-BR-VR, it was possible to see that both the full scale (49 items) and the reduced version (24 items) showed good reliability and convergent validity. The explanation coefficients were moderate for the factors: Working Conditions, Defense of Values and Rights, Ethical Infractions, and Safe and Qualified Care.

Overall, the study helped to highlight the importance of assessing MD in nurses, as it has a direct impact on their mental health and daily work, interfering in their actions and relationships, and the quality of patient care. Thus, the reduced MDSN-BR scale facilitates a clear, accessible approach, with a shorter response time, demonstrating reliability in all the items tested, which makes it a valid instrument for use in assessing nurses’ MD.

Já os fatores de reconhecimento, poder e identidade profissional evidenciaram graus fortes, o que implica em uma moderada capacidade de produzir previsões precisas e de replicabilidade em novos dados.

The limitations of this study are that it was only carried out in one hospital, making it impossible to compare it with other contexts.

## Conclusion

The validation of the MDSN-BR-VR achieved satisfactory psychometric indicators and reliability levels, proving to be suitable for use. This reduced version preserved the original structure of the scale, keeping the four items with the highest factor loadings per dimension, totaling 24 items.

The exploratory factor analysis of the MDSN-BR-VR, based on age invariance, provided a deeper understanding of the results and confirmed the validity of the reduced model. Therefore, both scales, the full version, and the reduced version showed similar behavior, giving researchers the freedom to choose the most appropriate instrument for their investigations.

However, the MDSN-BR-VR offers practical advantages, as it is easier to apply and understand, as well as requires less time for participants to respond. Finally, it is suggested that further research be carried out into moral distress, to deepen understanding of its consequences and develop effective strategies for managing it, to provide support for both professionals and healthcare organizations.

## References

[1] Ramos F. R. S., Brehmer L. C. F., Dalmolin G. L., Silveira L. R., Schneider D. G., Vargas M. A. O. (2020). Association between moral distress and supporting elements of moral deliberation in nurses. Rev. Latino-Am. Enfermagem.

[2] Becker R. P. (2024). The Impact of Moral Distress on Staff and Novice Nurses. J Christ Nurs.

[3] Laurs L., Blaževičienė A., Capezuti E., Milonas D. (2020). Moral Distress and Intention to Leave the Profession: Lithuanian Nurses in Municipal Hospitals. J Nurs Scholarsh.

[4] Emmamally W., Chiyangwa O. (2020). Exploring moral distress among critical care nurses at a private hospital in Kwa-Zulu Natal, South Africa. South Afr J Crit Care.

[5] Faraco M. M., Gelbcke F. L., Brehmer L. C. F., Ramos F. R. S., Barlem E. L. D., Dalmolin G. L. (2022). Moral distress experienced by nurse managers in the context of federal university hospitals. Acta Paul Enferm [Internet].

[6] Bruggmann M. S., Schneider D. G., Ramos F. R. S., Dalmolin G. L., Rodrigues J., Bhering A. (2023). Intensity and frequency of moral distress in mental health nurses in Brazil. Rev Esc Enferm USP.

[7] Maunder R. G., Heeney N. D., Greenberg R. A., Jeffs L. P., Wiesenfeld L. A., Johnstone J. (2023). The relationship between moral distress, burnout, and considering leaving a hospital job during the COVID-19 pandemic: a longitudinal survey. BMC Nurs.

[8] Ramos F. R. S., Barth P. O., Brehmer L. C. F., Dalmolin G. L., Vargas M. A., Schneider D. G. (2020). Intensity and frequency of moral distress in Brazilian nurses. Rev Esc Enferm USP.

[9] Ramos F. R. S., Barlen E. L. D., Brito M. J. M., Vargas M. A., Schneider D. G., Brehmer L. C. F. (2019). Validation of the Brazilian Moral Distress Scale in Nurses. J Nurs Meas.

[10] Anderson H., Birks Y., Adamson J. (2020). Exploring the relationship between nursing identity and advanced nursing practice: An ethnographic study. J Clin Nurs.

[11] Cardiff S., Gershuni O., Giesbergen-Brekelmans A. (2023). How local, first-line nurse leaders can positively influence nurse intent to stay and retention: A realist review. J Clin Nurs.

[12] Dey C., Ganesh M. P. (2020). Impact of team design and technical factors on team cohesion. Team Perform Manag.

[13] Atashzadeh-Shoorideh F., Tayyar-Iravanlou F., Chashmi Z. A., Abdi F., Cisic R. S. (2020). Factors affecting moral distress in nurses working in intensive care units: A systematic review. Clin Ethics.

[14] Han Y., Kim J. S., Seo Y. (2019). Cross-Sectional Study on Patient Safety Culture, Patient Safety Competency, and Adverse Events. Western J Nurs Res.

[15] Király P., Kiss R., Kovács D., Ballaj A., Tóth G. (2022). The Relevance of Goodness-of-fit, Robustness and Prediction Validation Categories of OECD-QSAR Principles with Respect to Sample Size and Model Type. Mol Inform.

[16] Lautert L. (1995). O desgaste profissional do enfermeiro [Dissertation].

[17] Lanes T. C., Dalmolin G. L., Silva A. M., Ramos F. R. S., Olson L. (2023). Cross-Cultural adaptation of the Hospital Ethical Climate Survey to Brazil. J Nurs Meas.

[18] Hair J. F., Sarstedt M, Ringle CM, Gudergan SP (2023). Advanced Issues in Partial Least Squares Structural Equation Modeling.

[19] Henseler J., Ringle C. M., Sarstedt M. (2016). Testing measurement invariance of composites using partial least squares. Int Mark Rev.

[20] Hu L., Bentler P. M. (1998). Fit Indices in Covariance Structure Modeling: Sensitivity to Underparameterized Model Misspecification. Psychol Methods.

[21] Fornell C., Larcker D. F. (1981). Evaluating Structural Equation Models with Unobservable Variables and Measurement Error. J Mark Res.

[22] Cohen J. (1988). Statistical Power Analysis for the Behavioral Sciences [Internet].

[23] Lopes L. F. D., Chaves B. M., Fabrício A., Porto A., Almeida D. M., Obregon S. L. (2020). Analysis of Well-Being and Anxiety among University Students. Int J Environ Res Public Health.

[24] Chin W., Cheah J. H., Liu Y., Ting H., Lim X. J., Cham T. H. (2020). Demystifying the role of causal-predictive modeling using partial least squares structural equation modeling in information systems research. Ind Manag Data Syst.

[25] Lopes L. F. D., Silva D. J. C., Kuhn N., Chiapinoto F. V., Lima M. P. (2024). The influence of technostress on anxiety disorder in higher education students during the Covid-19 pandemic. Knowl Manag E-learn.

[26] Laschinger H. K., Finegan J., Shamian J., Wilk P. (2001). Impact of structural and psychological empowerment on job strain in nursing work settings: expanding Kanter’s model. J Nurs Adm.

[27] Nejat N., Zand S., Taheri M., Khosravani M. (2023). Understanding lived experiences of nurse managers about managerial ethics. Nurs Ethics.

[28] Paige J. T., Garbee D. D., Bonanno L. S., Kerdolff K. E. (2021). Qualitative Analysis of Effective Teamwork in the Operating Room (OR). J Surg Educ.

[29] Donley J. (2021). The Impact of Work Environment on Job Satisfaction: Pre-COVID Research to Inform the Future. Nurse Lead.

[30] Woods M. (2020). Moral distress revisited: the viewpoints and responses of nurses. Int Nurs Rev.

[31] Ventovaara P., Sanderberg M. A., Blomgren K., Pergert P. (2023). Moral distress and ethical climate in pediatric oncology care impact healthcare professionals’ intentions to leave. Psychooncology.

[32] Manookian A., Nadali J., Ghiyasvandian S., Weaver K., Haghani S., Divani A. (2023). Spiritual care competence, moral distress and job satisfaction among Iranian oncology nurses. Int J Palliat Nurs.

[33] Morley G., Sankary L. R. (2023). Nurturing moral community: A novel moral distress peer support navigator tool. Nurs Ethics.

[34] Faraco M. M., Gelbcke F. L., Brehmer L. C. F., Ramos F. R. S., Schneider D. G. (2022). Moral distress-associated sociodemographic and occupational aspects in nursing managers at federal university hospitals. Rev Esc Enferm USP.

[35] Toescher A. M. R., Barlem E. L. D., Lunardi V. L., Brum A. N., Barlem J. G. T., Dalmolin G. L. (2020). Moral distress and professors of nursing: A cluster analysis. Nurs Ethics.

[36] Kolbe L., Melo-Martin I. (2023). Moral Distress: What Are We Measuring?. Am J Bioeth.

