# Supplementary material for: Validation of the reduced version of the Brazilian Scale of Moral Distress in Nurses
Source: Rev Lat Am Enfermagem. 2025 Feb 17;33:e4455. doi: 10.1590/1518-8345.7123.4455 (PMC11835009; doi:10.1590/1518-8345.7123.4455)
Supplement: Translation

# Translation: Validação da versão reduzida da Escala Brasileira de Distresse Moral em Enfermeiros

**DOI:** 10.1590/1518-8345.7123.4456

**Authors:** Jordana Lopes Carvalho, Luis Felipe Dias Lopes, Flavia Regina Souza Ramos, Graziele de Lima Dalmolin

**Affiliations:** 1 Universidade Federal de Santa Maria, Santa Maria, RS, Brasil; 2 Universidade Federal de Santa Maria, Departamento de Ciências Administrativas, Santa Maria, RS, Brasil; 3 Universidade Federal de Santa Catarina, Florianópolis, SC, Brasil; 4 Universidade Federal de Santa Maria, Departamento de Enfermagem, Santa Maria, RS, Brasil

**Keywords:** Enfermagem, Enfermeiras e Enfermeiros, Moral, Estresse Psicológico, Sistema Métrico, Estudo de Validação

## Abstract

validar um modelo da versão reduzida da Escala Brasileira de Distresse Moral em Enfermeiros.

estudo metodológico, com amostra de 269 enfermeiros de um hospital universitário público no Sul do Brasil. Foram utilizados um questionário sociodemográfico e a Escala Brasileira de Distresse Moral em Enfermeiros. Empregaram-se, para a análise estatística descritiva, a modelagem de equações estruturais e análise de invariância entre as faixas de idade.

quanto ao perfil, prevaleceram os profissionais na faixa etária de 31 a 40 anos, mulheres, casados, especialistas. O modelo estrutural se ajustou aos dados, com índices aceitáveis de ajuste. O modelo proposto apresentou excelente consistência interna, validade convergente e discriminante. A análise do modelo estrutural revelou que as cinco hipóteses foram confirmadas e a invariância entre as faixas de idade indica que o modo como as variáveis são conceitualizadas, medidas e inter-relacionadas no modelo não afetam os resultados, independentemente dos grupos comparados.

a escala alcançou indicadores psicométricos satisfatórios, mostrando-se adequada para o uso, mantendo os elementos de validação relevantes por dimensão e oferecendo vantagens práticas. Sugere-se a realização de novas pesquisas para aprofundar o entendimento sobre as consequências do distresse moral e desenvolver estratégias eficazes para seu manejo.

## Destaques

**(1)**A EDME-BR na versão reduzida ficou composta por 24 questões e seis fatores. **(2)**Empregada a análise de invariância e modelagem de equações estruturais para a validação. **(3)**Valores de consistência interna e confiabilidade composta foram adequados. **(4)**Os testes sinalizaram suficiente relevância preditiva do modelo proposto. **(5)**A escala reduzida propõe uma abordagem clara, acessível e com menor tempo de resposta.

## Introdução

No contexto da prática profissional da Enfermagem, o enfermeiro precisa conduzir as situações cotidianas que exigem decisões ético-morais, diante da complexidade do sistema de saúde. Estas decisões abarcam os aspectos organizacionais, estruturais, relacionados às equipes e aos usuários, e demandam posicionamentos e deliberações que suscitam nos profissionais uma gama de emoções – como inquietação, desconforto e incerteza frente aos conflitos. Tais circunstâncias podem culminar na vivência do Distresse Moral (DM)^(1)^.

Conceitua-se DM como a aflição interior associada aos conflitos exteriores entre os valores, obrigações e ações, e que se manifesta quando o enfermeiro reconhece a linha de conduta apropriada a seguir, porém, se vê impedido de agir de acordo com o próprio discernimento e julgamento^(1)^.

Dessa forma, as condições imprescindíveis e suficientes para definir o DM estão relacionadas à conciliação da experiência moral e de desordem emocional, em uma relação causal direta^(1)^. Na área da saúde, diante de diferentes circunstâncias eticamente frágeis, o DM tem-se destacado como um fenômeno ou experiência de grande relevância para a Enfermagem, devido à abrangência de causas e significados^(2-3)^.

Alguns estudos contemporâneos, no âmbito internacional, apontam níveis moderados de DM em enfermeiras hospitalares^(3-4)^. Essas descobertas estão em consonância com os resultados de um estudo conduzido com enfermeiras brasileiras atuantes no ambiente hospitalar, o qual relatou um nível moderado de frequência e intensidade de DM^(5-6)^. Porém, como agravante, o DM tem apresentado implicações que resultam em diminuição da satisfação no trabalho, desafios éticos, aumento da carga de trabalho e favorecem o aumento da rotatividade de profissionais^(7)^, o que demanda uma avaliação periódica das equipes e dos ambientes de trabalho.

Nesse contexto, o progresso das pesquisas demanda revisões contínuas e aprimoramentos nos instrumentos de mensuração, os quais desempenham um papel fundamental na avaliação do sofrimento moral entre os profissionais de saúde^(8)^. Atualmente, o DM é avaliado por meio da Escala Brasileira de Distresse Moral em Enfermeiros (EDME-BR), que contempla as peculiaridades da profissão e dos contextos da prestação de serviços no Brasil^(9)^.

Este instrumento evidenciou a presença de DM com diversos fatores preditivos, apontou as similaridades entre as experiências dos enfermeiros no contexto nacional e internacional, além de abordar o cenário assistencial em diferentes níveis de complexidade e reconhecer as particularidades do DM no Brasil^(9)^.

Ao considerar o exposto, reafirma-se a importância de mensurar a intensidade e a frequência do DM como um instrumento apropriado ao ambiente de trabalho e cenário político-profissional, com foco na otimização do tempo de resposta e para facilitar a avaliação nos diferentes contextos de atuação. Justifica-se tal necessidade, na particularidade da EDME-BR, por se tratar de uma escala composta por 49 itens, a qual foi empregada em estudo no grupo de pesquisa com 269 enfermeiros, quando se reconheceu o elevado tempo de resposta e muitas recusas de participação por ser um instrumento longo. Assim, visualizou-se a possibilidade, com a utilização do banco de dados do estudo, de realizar nova análise metodológica para reduzir a escala. A relevância disso está associada ao fato de que o maior conhecimento sobre o DM, utilizando uma escala com mais facilidade de uso, pode contribuir significativamente para a compreensão dos trabalhadores sobre suas circunstâncias e os desafios cotidianos.

Diante disso, apresentou-se como questão de pesquisa: “A EDME-BR apresenta validade e confiabilidade adequadas em um modelo reduzido?”. Para respondê-la, obteve-se como objetivo validar um modelo da versão reduzida da EDME-BR com profissionais de uma instituição hospitalar pública da Região Sul do Brasil.

Destarte, apresentaram-se como hipóteses para a redução da escala as possíveis relações entre as dimensões da escala, com a posterior contextualização:

H1: A percepção de Reconhecimento, Poder e Identidade Profissional (RPIP) está relacionada à capacidade de Defesa de Valores e Direitos (DVD);

H2: O reconhecimento, poder e identidade profissional dos enfermeiros estão relacionados com a eficácia e coesão das Equipes de Trabalho (ET);

H3: As Condições de Trabalho (CT) têm um impacto direto na percepção sobre as ET;

H4: As CT (adversas) estão relacionadas à percepção e à ocorrência de infrações éticas no ambiente de Enfermagem;

H5: Há relação entre a capacidade de fornecer Cuidado Seguro e Qualificado (CSQ) e a percepção de Infrações Éticas (IEs);

H6: A análise de invariância entre as faixas de idade dos enfermeiros supõe que não existe diferença entre os enfermeiros com até 35 anos e os enfermeiros com idade acima de 35 anos para as cinco hipóteses testadas.

Por fim, as hipóteses apresentadas estão de acordo com a literatura, visto que: a H1 sugere que os enfermeiros que se sentem mais reconhecidos e seguros em sua identidade profissional estão mais inclinados a defender os valores e os direitos no ambiente de trabalho^(10)^; a H2 sugere que os enfermeiros que percebem um maior reconhecimento de suas competências e valor profissional tendem a contribuir de forma mais efetiva para o trabalho em equipe, promovendo um ambiente colaborativo e de suporte mútuo^(11)^; a H3 sugere que as melhores condições de trabalho promovem uma melhor dinâmica e coesão de equipe^(12)^; a H4 sugere que os ambientes de trabalho com recursos insuficientes, alta carga de trabalho, pressão intensa e suporte organizacional inadequado aumentam o risco de comprometimento ético, levando à tomada de decisões que podem não estar em conformidade com os padrões éticos e profissionais^(13)^; e, por último, H5 indica que maiores competência e segurança no cuidado estão associadas à menor percepção de infrações éticas no ambiente de trabalho^(14)^.

## Método

### Delineamento do estudo

Trata-se de um estudo metodológico, com foco na validação de um instrumento a partir de modelagem estatística, com a utilização de banco de dados do projeto: “Sofrimento moral em enfermeiros hospitalares: qual sua relação com clima ético e *Burnout*?”, registrado no Portal de Projetos da universidade de origem da pesquisadora responsável. No método de análise citado, os testes de qualidade de ajuste relacionam-se com quão bem um modelo se ajusta aos dados. Esses testes auxiliam a determinar se o modelo escolhido reproduz as variáveis resposta nas quais os parâmetros foram otimizados, ou se é necessário considerar um modelo distinto^(15)^.

### Cenário

O estudo foi desenvolvido em um hospital universitário público na Região Sul do Brasil. Atualmente a instituição possui 400 leitos e atua como referência na região pela tecnologia e nível de atendimentos de média e alta complexidade que possui, apoiado pela ampla equipe de trabalhadores e estudantes da área.

### População, critérios de seleção e definição da amostra

Os participantes foram 269 enfermeiros que participaram do projeto matricial citado e que compuseram o banco de dados do mesmo. O projeto matricial tinha como população 303 enfermeiros que atuavam na instituição no momento da coleta de dados, valor que foi tomado como base para o emprego de fórmula para o cálculo da amostra para a população finita, juntamente com erro amostral de 5% e percentual estimado de 50%; o que resultou em um mínimo de 171 participantes. Como critérios de inclusão, foram selecionados os enfermeiros com no mínimo um mês de trabalho (tempo mínimo estimado para a percepção sobre o ambiente de trabalho). Como critério de exclusão, elencaram-se: enfermeiros que se encontravam em licença ou afastamento do trabalho durante o período da coleta de dados.

### Instrumentos utilizados para a coleta das informações e as variáveis do estudo

O banco de dados do projeto matricial foi composto por um questionário sociodemográfico e laboral, a escala EDME-BR, o Inventário de *Burnout*de Maslach^(16)^ e o *Hospital Ethical Climate Survey*(HECS)^(17)^. Porém, para este estudo foram utilizadas as variáveis do questionário sociodemográfico e laboral, e a escala EDME-BR^(9)^. O questionário sociodemográfico e laboral abordou as seguintes variáveis: sexo, estado civil, idade (transformada em duas faixas de idade, isto é, até 35 anos e acima de 35 anos), formação, tempo de formação, turnos de trabalho e carga horária.

A EDME-BR^(9)^ é um instrumento construído e validado no Brasil em 2019, o qual resultou em uma versão final da escala com 49 itens distribuídos em seis fatores, para os quais os valores de alfa de Cronbach variaram de 0,91 a 0,96^(9)^. Estes são: Fator 1 - RPIP, com 11 questões (14, 15, 16, 34, 35, 36, 37, 38, 40, 41, 43); Fator 2 - CSQ, com 11 questões (23, 24, 25, 26, 27, 28, 29, 30, 31, 32, 33); Fator 3 - DVD, com oito questões (39, 42, 44, 45, 46, 47, 48, 49); Fator 4 - CT, com seis questões (8, 9, 10, 11, 12, 13); Fator 5 - IEs, com seis questões (17, 18, 19, 20, 21, 22); e pelo Fator 6 - ET, com sete questões (1, 2, 3, 4, 5, 6, 7). Para cada item implementa-se escala Likert de sete pontos (de 0 = nenhum/nunca a 6 = muito intenso/muito frequente).

### Período e coleta de dados

O acesso ao banco de dados foi realizado em maio de 2022. A coleta de dados do projeto matricial foi conduzida entre abril e junho de 2019. Foi realizada pelos autores e membros do grupo de pesquisa que não possuíam proximidade com os participantes, e que foram previamente treinados e obtiveram a experiência em coleta de dados quantitativos.

Os enfermeiros foram convidados a participar da pesquisa em seus locais de trabalho, optando por responder o instrumento de imediato ou entregá-lo posteriormente, após a assinatura de Termo de Consentimento Livre e Esclarecido (TCLE), o qual foi assinado tanto pelo participante quanto pelo pesquisador, sendo uma cópia entregue a cada um.

Todos os enfermeiros que aceitaram participar da pesquisa receberam as informações detalhadas sobre os objetivos e os procedimentos, potenciais riscos e os benefícios envolvidos, além da garantia do direito de retirarem seu consentimento para participar do estudo a qualquer momento, sem qualquer repercussão negativa ou exposição pública de suas informações.

No caso de entrega posterior dos instrumentos preenchidos pelos profissionais, a busca foi agendada e foram feitas até três tentativas para recuperar os instrumentos preenchidos, em datas e momentos distintos. Ainda, durante o processo de preenchimento, os coletadores garantiram a liberdade dos participantes ao se aproximarem apenas no final, para coletar os instrumentos, ou para esclarecer as eventuais dúvidas.

Para garantir a confidencialidade das informações, os formulários preenchidos foram armazenados pelos coletadores em envelopes lacrados, entregues ao pesquisador responsável.

### Tratamento e análise dos dados

O banco de dados foi constituído inicialmente no programa *EpiInfo* ^®^(versão 6.4), a partir de dupla digitação independente, verificação dos erros e inconsistências. As variáveis e as escala selecionadas no banco de dados foram transportadas para a análise nos *softwaresSmartPLS* ^®^(versão 4.1.0.1) e *Statistical Package for the Social Sciences*(SPSS^®^versão 26). As variáveis sociodemográficas e laborais foram analisadas por meio de estatística descritiva, com distribuição de frequência absoluta (N) e relativa (%) para as variáveis categóricas.

Para validar a escala reduzida, utilizou-se a técnica de modelagem de equações estruturais, pelo método de mínimos quadrados parciais, e a Análise Multigrupo (MGA), mais especificamente, a Análise de Invariância. O modelo escolhido atendeu à finalidade de avaliar a estrutura de inter-relações entre as dimensões da escala proposta e uma já validada, por meio da testagem das hipóteses propostas^(18)^. Ainda, na análise dos dados utilizou-se da estimação modelo de caminho *Partial Least Squares - Structural Equation Modeling*(PLS-SEM)^(16)^.

Este estudo adotou como critérios para avaliar o ajuste do PLS-SEM o *Standardized Root Mean Square Residual*(SRMR), a distância euclidiana quadrada (dDEQ), a distância geodésica (dG) e o *Normed Fit Index*(NFI). Adotou-se 0,08 como valor limiar de SRMR^(19)^ e 0,8 como valor mínimo de NFI^(20)^.

O modelo de mensuração da EDME-BR apresentou cinco hipóteses diretas, as quais se conectam em três dimensões (dimensões exógenas) com três dimensões endógenas (preditivas) contendo 24 itens (variáveis observadas) (Tabela 1). Tais hipóteses oportunizam avaliar a invariância da escala reduzida entre as faixas de idade, afirmando que a escala possa mensurar aquilo a que se destina.

Somado a isso, a Análise Fatorial Confirmatória (AFC) comparativa entre as faixas de idade foi realizada como parte integrante da avaliação do PLS-SEM, com vistas a confirmar e a refinar os itens e dimensões dispostos no modelo. A consistência interna entre os componentes foi verificada, em cada dimensão, a partir do alfa de Cronbach (AC). A validade discriminante foi apurada utilizando os critérios Fornell-Larcker (FL) e *Heterotrait-Monotrait Ratio* (HTMT), para determinar em que medida uma dimensão dentro de seus componentes diferiu de outra^(21)^.

A raiz quadrada do valor da Variância Média Extraída (VME) (Tabela 3) de cada dimensão foi medida, comparada com os valores da Matriz de Correlação e do critério HTMT, utilizando o procedimento de *bootstrap*, o qual possuiu valores menores que um para o limite superior (97,5% de confiança).

Também nessa etapa, foram verificadas a confiabilidade de consistência interna, validade convergente e discriminante^(20)^, ou seja, contemplou-se a assunção da Variância Média Extraída (AVE > 0,5), o alfa de Cronbach (0,7 < AC < 0,95) e a confiabilidade composta (0,7 < CC < 0,95) para o modelo^(22- 23)^. E, para avaliar se o modelo estrutural representou as teorias subjacentes ao modelo de mensuração, permitiu-se analisar a capacidade preditiva do modelo e as relações entre as dimensões desse modelo, e foi considerada a análise de (multi)colinearidade estimando o *Variance Inflation Factor* (VIF), que possuiu valores para as variáveis latentes inferiores a 5^(22)^.

Para avaliar a significância dos valores dos coeficientes de caminho nas relações diretas entre os fatores, com base no valor *t* de *Student*, utilizou-se a técnica de *bootstrapping* com 5.000 subamostras. A relação direta entre os fatores foi aferida a partir de coeficientes estruturais (β’s), e a relação de caminho considerada significativa, ao se obter níveis de significância de 5%, com valor *t* inferior a 1,96^(18)^.

Quanto ao procedimento de redução, foram empregadas as técnicas de avaliação do modelo estrutural, com vistas a verificar sua consistência interna e validade convergente. Na avaliação da validade discriminante do modelo, optou-se por considerar as cargas fatoriais (λ) mais expressivas para cada uma das dimensões. Em seguida, o modelo foi submetido a uma avaliação de sua estrutura, incluindo a análise da precisão (R^2^) e da relevância preditiva (Q^2^)^(18)^.

Uma etapa crucial na avaliação de um modelo PLS-SEM é a testagem da precisão e a relevância preditiva dele. A acurácia preditiva foi testada por meio do coeficiente de determinação (R^2^) e o respectivo valor de p, que fornece o grau de variância explicado em cada dimensão endógena^(18)^. O valor de R^2^ varia entre 0 e 1, quanto maior o seu valor, maior a acurácia preditiva^(18)^. Além disso, a significância de R^2^ também foi avaliada, com valores indicando diferentes níveis de acurácia preditiva: 0,075 < R^2^ ≤ 0,19 (efeito moderado) e R^2^> 0,19 (efeito forte)^(22- 23)^.

A relevância preditiva foi avaliada por meio do procedimento de venda nos olhos, que calculou o valor de Q^2^ , utilizando a técnica de *blindfolding* . Um valor de Q^2^ superior a 0 é considerado indicativo de relevância preditiva^(18-23)^. Os valores de Q^2^ variam entre 0,075 e 0,25 para um grau moderado de relevância preditiva, enquanto Q2 > 0,25 é indicativo de um grau forte de relevância preditiva^(23- 24)^.

### Aspectos éticos

Foram respeitados todos os preceitos éticos da pesquisa envolvendo seres humanos, conforme a Resolução nº 466/12, com o projeto aprovado por Comitê de Ética em Pesquisa (CEP) local sob Parecer nº 2.764.702.

## Resultados

### Perfil dos participantes

Participaram da pesquisa 269 enfermeiros, dentre os quais 83 (30,9%) tinham até 35 anos e 186 (69,1%), acima de 35 anos. A maioria dos participantes era do sexo feminino (88,1%), e 75,09% eram casados. Quanto à formação acadêmica, 54,37% dos participantes possuíam especialização e 53,17% haviam concluído sua formação entre seis e 15 anos antes da pesquisa. Em relação à carga horária de trabalho, a maioria dos enfermeiros (65,06%) trabalhava 30 horas ou mais por semana, e 39,04% possuíam turnos de trabalho mistos. Destaca-se que, ao se considerar que o tempo de atuação no hospital era um critério de inclusão, a mediana do tempo de trabalho na instituição foi de quatro (AIq10) anos, 187 (69,5%) tinham quatro anos ou mais, e apenas quatro (1,49%) apresentavam um mês de trabalho, enquanto todos os demais tinham acima de seis meses de atuação.

### Testes de ajuste do modelo

Os resultados confirmaram que o modelo estrutural sugerido se ajustou aos dados após oito interações com índices aceitáveis, com SRMR = 0,062, d_DEQ_ = 2,563, d_G_ = 1,605, e NFI = 0,848.

### Avaliação do modelo de medição

Nessa etapa foram avaliados os três critérios: confiabilidade de consistência interna, validade convergente e discriminante, contemplando a variância média extraída.

A consistência interna entre os componentes, em cada dimensão, foi verificada por meio do AC e CC. Os valores de AC de todas as dimensões variaram de 0,798 a 0,930. Além disso, os valores de Confiabilidade Composta (CC) variaram de 0,817 a 0,947. Por fim, as VMEs apresentaram valores entre 0,701 e 0,950. Quanto às cargas fatoriais, para a escala EDME-BR-R os valores das cargas fatoriais foram superiores a 0,6 (Figura 2), portanto, esses resultados afirmaram a garantia de consistência interna entre os indicadores e seus respectivos fatores. A Tabela 1 apresenta os valores da avaliação de todas as dimensões do modelo de medição.

Ao avaliar o modelo quanto à validade discriminante, foram utilizados, como principais critérios, o FL e o HTMT. O critério FL comparou a raiz quadrada do valor da VME de cada dimensão medida aos valores da matriz de correlação. Observou-se que a menor VME (0,802) é superior à maior correlação CT vs. CSQ (0,679), portanto, o critério foi estabelecido. Já o critério HTMT determinou que os limites superiores estimados foram inferiores a 1.0. Assim, verificou-se que o modelo apresenta validade discriminante e, desse modo, satisfez os requisitos.

Em resumo, as avaliações do modelo de mensuração quanto à consistência interna, validade convergente e discriminante atenderam aos seus requisitos, validando empiricamente a adequação do modelo de mensuração neste estudo (Tabelas 1 e 2).

**Tabela 1 -:** Avaliação do modelo de mensuração – consistência interna e validade convergente. Santa Maria, RS, Brasil, 2019

EDME-BR* (Dimensões)	**AC** ^†^		**CC** ^‡^		**VME** ^§^	
	**≤ 35** ^ǁ^	**> 35** ^¶^	**≤ 35** ^ǁ^	**> 35** ^¶^	**≤ 35** ^ǁ^	**> 35** ^¶^
RPIP**	0,857	0,798	0,904	0,817	0,701	0,865
DVD^††^	0,875	0,883	0,912	0,904	0,721	0,919
IEs^§§^	0,898	0,930	0,929	0,936	0,766	0,950
CSQ^‖‖‖^	0,925	0,899	0,947	0,900	0,816	0,930
CTs^¶¶^	0,868	0,825	0,909	0,835	0,714	0,882
ETs***	0,785	0,830	0,861	0,838	0,608	0,887

*EDME-BR = Escala Brasileira de Distresse Moral em Enfermeiros;^†^AC = Alfa de Cronbach;^‡^CC = Confiabilidade Composta;^§^VME = Variância Média Extraída; **^ǁ^** ≤ 35 = Menor e igual a 35 anos;^¶^> 35 = Maior que 35 anos; **RPIP = Reconhecimento, Poder e Identidade Profissional;^††^DVD = Defesa dos Valores e Direitos;^§§^IEs = Infrações éticas;^ǁǁ^CSQ = Cuidado Seguro e Qualificado;^¶¶^CTs = Condições de Trabalho; ***ETs = Equipes de Trabalho

**Tabela 2 -:** Critério Fornell-Larcker e HTMT* do modelo fatorial. Santa Maria, RS, Brasil, 2019

**Dim.** ^†^	^‡^	**Matriz de Correlação de Pearson**					
		**RPIP** ^§^	**DVD** ^‖‖^	**IEs** ^¶^	**CSQ** ^**^	**CTs** ^††^	**ETs** ^‡‡^
RPIP^§^	0,821	1,000					
DVD^‖‖^	0,885	0,632	1,000				
IEs^¶^	0,856	0,476	0,607	1,000			
CSQ**	0,805	0,586	0,527	0,561	1,000		
CTs^††^	0,897	0,555	0,664	0,674	0,679	1,000	
ETs^‡‡^	0,802	0,551	0,576	0,575	0,665	0,647	1,000
		Limite Superior (HTMT*)_97,5%_					
DVD^‖‖^		0,733					
IEs^¶^		0,811	0,816				
CSQ**		0,742	0,758	0,790			
CTs^††^		0,740	0,648	0,707	0,821		
ETs^‡‡^		0,866	0,738	0,873	0,709	0,785	

*HTMT =*Heterotrait-Monotrait Ratio*;^†^Dim. = Dimensão;^‡^= Raiz da Variância Média Extraída;^§^RPIP = Reconhecimento, Poder e Identidade Profissional;^‖‖^DVD = Defesa dos Valores e Direitos;^¶^IEs = Infrações Éticas; **CSQ = Cuidado Seguro e Qualificado;^††^CTs = Condições de Trabalho;^‡‡^ETs = Equipes de Trabalho

### Análise do modelo estrutural

Após analisar a colinearidade entre as dimensões e os valores da VIF terem sido calculados entre 1,000 < VIF < 1,664, o modelo não apresentou fortes correlações, portanto, os valores dos coeficientes de regressão (β’s) foram estimados utilizando a técnica*bootstrapping*para 5.000 subamostras. Os valores e suas significâncias estão descritos na Tabela 3.

**Tabela 3 -:** Resultados de efeitos diretos entre dimensões. Santa Maria, RS, Brasil, 2019

**Relação de caminho (Hipóteses)**		**Coeficientes de caminho**		**Desvio-Padrão**	Estat. t.*	**p-valor** ^†^	**Resultado**
H1^‡^	RPIP^§^→ DVD^‖‖^	0,575	0,040		14,526	0,000	Suporta
H2^¶^	RPIP^§^→ ETs**	0,492	0,056		8,763	0,000	Suporta
H3^††^	CTs^‡‡^→ ET**	0,315	0,059		5,288	0,000	Suporta
H4^§§^	CT^‡‡^→ IEs^‖‖‖‖^	0,226	0,062		3,652	0,000	Suporta
H5^¶¶^	CSQ*** → CT^‡‡^	-0,521	0,056		9,349	0,000	Suporta

*Estat. t = Teste t de Student;^†^p-valor = Significância;^‡^H1 = Hipótese 1;^§^RPIP = Reconhecimento, Poder e Identidade Profissional;^ǁ^DVD = Defesa dos Valores e Direitos;^¶^H2 = Hipótese 2; **ETs = Equipes de Trabalho;^††^H3 = Hipótese 3;^‡‡^CTs = Condições de Trabalho;^§§^H4 = Hipótese 4;^ǁǁ^IEs = Infrações Éticas;^¶¶^H5 = Hipótese 5; ***CSQ = Cuidado Seguro e Qualificado

Todas as hipóteses propostas confirmaram relações significativas (p < 0,05) entre as dimensões, portanto, as hipóteses foram empiricamente sustentadas a partir dos valores de t para um nível de significância de 5%. Observou-se que o cuidado seguro e qualificado se relaciona negativamente com as infrações éticas, dez relações foram significativas (Figura 1).

A análise (Figura 1) revelou que o modelo exibiu dimensões com coeficientes de determinação (R^2^) indicando graus fortes de explicação (R^2^ > 0,19) para as variáveis preditivas, isso afirma que esses resultados foram consistentes. Além disso, os valores de relevância preditiva das dimensões endógenas, estimados por meio do método de *blindfolding*, demonstraram valores de Q ^2^ superiores a 0, indicando uma boa capacidade de predição do modelo. Mais especificamente, os valores de Q ^2^ variaram de 0,323 a 0,498.

A seguir, naTabela 4, apresenta-se a análise de invariância do modelo, referindo-se à Hipótese H6, onde compara as hipóteses H1 a H5 entre as faixas de idade até 35 anos, em comparação à faixa de idade acima de 35 anos.

Observou-se, na Tabela 4, que, tanto para os testes de Henseler, quanto para o teste de permutações, não há diferença significativa entre os coeficientes das diferentes faixas de idade (p > 0,05). Portanto, conclui-se que o modelo proposto não apresenta problemas de invariância e, assim, a escala se comporta de maneira consistente entre os grupos^(25)^.

**Figura 1 -:** Modelo de equações estruturais final. Santa Maria, RS, Brasil, 2019

*RPID = Reconhecimento, Poder e Identidade Profissional;^†^DVD = Defesa dos Valores e Direitos;^‡^CTs = Condições de Trabalho;^§^ETs = Equipes de Trabalho;^ǁ^CSQ = Cuidado Seguro e Qualificado;^¶^IEs = Infrações Éticas

**Tabela 4 -:** Análise multigrupo (MGA*) pelos testes de Henseler’s e Permutações. Santa Maria, RS, Brasil, 2019

**Hip.** ^‡^	**Relação**	**Coeficientes Estruturais**			**p-valor** ^†^ **(diferenças)**		**Resultado**
		**At.35** ^§^	**Ac.35** ^ǁ^	**At-Ac** ^¶^	Henseler’s MGA**	**Teste de Permutação**	
H1^††^	RPIP^‡‡^→ DVD^§§^	0,640	0,549	0,090	0,283	0,306	Não / Não
H2^ǁǁ^	RPIP^‡‡^→ ETs^¶¶^	0,426	0,516	-0,091	0,429	0,469	Não / Não
H3***	CTs^†††^→ ETs^¶¶^	0,371	0,312	0,059	0,625	0,642	Não / Não
H4^‡‡‡^	CTs^†††^→ IEs^§§§^	0,189	0,245	-0,057	0,679	0,688	Não / Não
H5^ǁǁǁ^	CSQ^¶¶¶^→ CTs^†††^	-0,620	-0,477	-0,143	0,245	0,222	Não / Não

*MGA =*Multi Group Analysis*;^†^p-valor = Significância;^‡^Hipóteses;^§^At. 35 = até 35 anos;^ǁ^Ac. 35 = Acima de 35 anos;^¶^At – Ac = Diferença entre até e acima de 35 anos;^††^H1 = Hipótese 1;^‡‡^RPIP = Reconhecimento, Poder e Identidade Profissional;^§§^DVD = Defesa dos Valores e Direitos;^ǁǁ^H2 = Hipótese 2;^¶¶^ETs = Equipes de Trabalho; ***H3 = Hipótese 3;^†††^CTs = Condições de Trabalho;^‡‡‡^H4 = Hipótese 4;^§§§^IEs = Infrações Éticas;^ǁǁǁ^H5 = Hipótese 5;^¶¶¶^CSQ = Cuidado Seguro e Qualificado

O alfa de Cronbach para o modelo EDMER-BR apresentou boa confiabilidade em todos seus fatores (0,95), valor menor que o da versão original (0,98). A versão reduzida da EDME-BR, contendo 24 itens, foi obtida a partir do cálculo dos índices de confiabilidade (AC e CC), considerando, como critério de seleção dos indicadores, os quatro itens por dimensão com maior carga fatorial.

Ainda, de forma a preservar as características da escala, as alterações realizadas não modificaram as instruções e escala de respostas ou interferiram na qualidade dos resultados obtidos. Portanto, a EDME-BR, na versão reduzida, ficou composta por 24 questões que avaliam os seguintes fatores:

Fator 1 - Reconhecimento, Poder e Identidade Profissional (RPIP), com quatro questões: 14) Sentir-se discriminado em relação a outros profissionais; 36) Trabalhar sob pressão pela insuficiência de tempo para o alcance de metas ou realização de tarefas; 40) Sentir-se desrespeitado por superiores hierárquicos; 41) Reconhecer atitudes eticamente incorretas dos gestores ou superiores hierárquicos.

Fator 2 - Cuidado Seguro e Qualificado (CSQ), com quatro questões: 24) Reconhecer que o acolhimento do usuário é inadequado; 25) Reconhecer que as demandas de continuidade do cuidado do paciente/usuário não são atendidas; 26) Reconhecer a falta de resolutividade das ações de saúde por causa dos problemas sociais; e 28) Reconhecer que as ações educativas com o usuário são insuficientes.

Fator 3 - Defesa dos Valores e Direitos (DVD), com quatro questões: 39) Vivenciar as condutas assistenciais que desconsideram as crenças e a cultura dos pacientes; 42) Sentir-se pressionado a pactuar ou silenciar frente às fraudes em benefício da instituição; 45) Reconhecer as situações de desrespeito/maus-tratos por parte dos profissionais em relação ao usuário; e 46) Reconhecer as situações de desrespeito ao direito do usuário à privacidade/intimidade.

Fator 4 - Condições de trabalho (CTs), com quatro questões: 8) Reconhecer que os materiais de consumo são insuficientes; 10) Reconhecer que os equipamentos/materiais permanentes disponíveis são insuficientes; 12) Reconhecer que a estrutura física do serviço é insuficiente; e 13) Reconhecer que a estrutura física do serviço é inadequada.

Fator 5 - Infrações Éticas (IEs), com quatro questões: 17) Vivenciar a omissão por parte do médico; 18) Vivenciar a imprudência por parte do médico; 19) Vivenciar a omissão por parte do enfermeiro; e 20) Vivenciar a imprudência por parte do enfermeiro.

Fator 6 - Equipes de trabalho (ETs), com quatro questões: 1) Trabalhar com número insuficiente de profissionais para a demanda; 3) Vivenciar as condições de sobrecarga de trabalho; 5) Trabalhar com enfermeiros despreparados; e 6) Trabalhar com auxiliares e técnicos de Enfermagem despreparados.

Para cada item implementa-se a escala Likert de sete pontos (de 0 = nenhum/nunca a 6 = muito intenso/muito frequente).

## Discussão

A partir dos resultados foi possível validar a EDME-BR na sua versão reduzida, sendo denominada “Escala Brasileira de Distresse Moral em Enfermeiros – Versão reduzida” (EDME-BR-VR), a qual apresentou uma redução de 25 questões, comparada à versão original. A EDME-BR-VR permaneceu com 24 questões, mantendo os fatores, isto é: Reconhecimento, Poder e Identidade Profissional; Cuidado Seguro e Qualificado; Defesa dos Valores e Direitos; Condições de Trabalho; Infrações Éticas; e Equipes de Trabalho. Porém, agora, cada um composto por quatro itens apenas.

Nesse modelo, o estudo obteve SRMR = 0,062 e NFI = 0,848. A raiz quadrada média dos resíduos padronizados é uma medida de ajuste que objetiva avaliar o grau de adequação do modelo, devendo ser inferior a 0,08 para o resultado satisfatório^(19-20)^. Já o NFI é a razão entre o χ² do modelo independente e o χ² do modelo que foi testado, não penaliza a complexidade do modelo, e é sensível ao tamanho da amostra. É uma comparação entre a matriz de covariância prevista pela AFC e a matriz original do que é previsto pelo modelo, devendo ser superior a 0,8^(19-20)^.

Quanto às hipóteses apresentadas e constatadas, a relação entre RPIP e DVD pode ser explicada pelo conceito de “*empowerment*” profissional, que envolve ter o poder, autoridade e capacidade para executar as ações baseadas em valores éticos e profissionais. Enfermeiros que percebem um maior “*empowerment*” no trabalho sentem-se mais capacitados para agir de acordo com seus valores profissionais, o que inclui defender os direitos dos pacientes e aderir aos padrões éticos elevados^(26-27)^.

A identidade profissional forte e o reconhecimento no ambiente de trabalho contribuem para a melhor dinâmica de equipe. A clareza do papel e o reconhecimento das habilidades dos enfermeiros estão associados à melhor comunicação e à colaboração entre os membros da equipe, facilitando a coesão e a eficácia do grupo^(28)^.

A influência das condições de trabalho na percepção das equipes de Enfermagem pode ser compreendida por meio da teoria dos recursos do trabalho, que postula que os ambientes de trabalho com recursos funcionais melhoram a satisfação e o engajamento dos funcionários, além de promover as relações positivas entre os colegas. As melhores condições de trabalho estão associadas à melhor qualidade de cuidados e à satisfação no trabalho entre os enfermeiros^(29)^.

Sabe-se que as condições de trabalho adversas podem criar dilemas éticos, onde os enfermeiros se veem forçados a fazer escolhas que comprometem os padrões éticos. Ambientes estressantes e com recursos insuficientes aumentam os conflitos éticos enfrentados pelos enfermeiros, evidenciando a relação entre as condições de trabalho e a ocorrência de infrações éticas^(30)^.

A capacidade de fornecer um cuidado seguro e qualificado pode ser comprometida em ambientes onde prevalecem as infrações éticas, criando uma relação negativa entre estas dimensões. Ou seja, os impactos decorrentes do DM podem comprometer o desempenho dos enfermeiros e influenciar negativamente a eficácia no cumprimento dos objetivos de saúde estabelecidos^(13)^.

O fato de todas essas hipóteses não diferirem entre as faixas etárias até 35 anos e acima de 35 anos sugere uma universalidade nas percepções e nas experiências dos enfermeiros, independentemente da idade. Isso pode indicar que as estruturas profissionais, éticas e organizacionais são percebidas de maneira consistente ao longo da carreira de Enfermagem, reforçando a importância de abordar essas dimensões em estratégias de desenvolvimento profissional e políticas de saúde.

Um estudo realizado, no contexto internacional, com profissionais de Enfermagem, evidenciou os níveis significativos na intensidade e na frequência de sofrimento moral frente às condições de trabalho insuficientes^(31)^.

As hipóteses de realização profissional e DM não tiveram implicação entre seus escores e corroboraram os achados de um estudo realizado com 280 enfermeiros, que verificou a relação da satisfação no trabalho e o sofrimento moral. A satisfação é um constructo inverso ao DM, ou seja, quanto maior o grau de DM, menor será a realização profissional^(32)^.

Escores de sofrimento moral e satisfação profissional demonstraram uma correlação negativa entre suas variáveis, apontando que esses constructos são inversamente proporcionais^(33)^. Níveis de realização profissional podem estar associados aos aspectos organizacionais, de interações, de autonomia e de remuneração, que, em níveis baixos, podem ocasionar manifestações físicas, devidas à sobrecarga de trabalho, ou emocionais, como estresse, ansiedade, frequentemente geradores da insatisfação no trabalho e do sofrimento moral^(7)^.

Os sentimentos de desvalorização e impotência por parte dos profissionais podem ocasionar conflitos éticos/morais, levando à insatisfação no trabalho^(33)^. Contudo, foi possível perceber, na versão reduzida da escala, que as maiores médias de DM se vinculam aos constructos cuidado seguro e qualificado, condições de trabalho, seguidos de infrações éticas.

Esses resultados convergem com outros estudos realizados com a aplicação da escala original. Um estudo com enfermeiros hospitalares demonstrou os maiores índices de sofrimento moral relacionados aos construtos “cuidado seguro e qualificado”, “condições de trabalho” e “equipes de trabalho”^(34)^.

Ainda, dos 269 enfermeiros participantes, na maioria do sexo feminino, obteve-se a prevalência de faixa etária de 31 a 40 anos. Um estudo que verificou DM em enfermeiros obteve resultados similares, com perfil representado majoritariamente por mulheres, com idade média de 37 anos^(35)^.

A ocorrência de DM associa-se à natureza subjetiva do trabalho, à experiência profissional e às condições organizacionais, especialmente com os desafios enfrentados no cotidiano laboral^(8)^. Um evento que desencadeie o DM, seja ele frequente ou esporádico, pode fornecer dados importantes que devem ser analisados, com destaque para a relevância das circunstâncias que o envolvem.

Embora as diversas definições de DM tenham sido sugeridas pela literatura, há a concordância de que ele emerge de limitações inadequadas na prática clínica que impactam a autonomia moral dos profissionais de saúde. Assim, para mitigar o sofrimento moral, é crucial que os instrumentos de medição forneçam as informações relevantes sobre essas limitações^(36)^, sendo a EDME-BR-VR uma opção válida e confiável.

Ao comparar os resultados obtidos na EDME-BR e na EDME-BR-VR, foi possível perceber que, tanto a escala em sua versão completa (49 itens), quanto em sua versão reduzida (24 itens) apresentaram boa confiabilidade e validade convergente. Os coeficientes de explicação apresentaram-se com graus moderados para os fatores: Condições de Trabalho, Defesa dos Valores e Direitos, Infrações Éticas e Para Cuidado Seguro e Qualificado.

De modo geral, o estudo contribuiu para destacar a importância de avaliar o DM em enfermeiros, pois ele repercute diretamente na saúde mental e no cotidiano de seu trabalho, interferindo nas suas ações e relações, e na qualidade do cuidado ao paciente. Assim, a escala EDME-BR reduzida facilita uma abordagem clara, acessível, com menor tempo de resposta, demonstrando uma confiabilidade em todos os itens testados, o que a torna um instrumento válido para ser utilizado na avaliação de DM de enfermeiros.

Já os fatores de reconhecimento, poder e identidade profissional evidenciaram graus fortes, o que implica em uma moderada capacidade de produzir previsões precisas e de replicabilidade em novos dados.

As limitações deste estudo devem-se a ele ter sido realizado somente em uma instituição hospitalar, impossibilitando a comparação com outros contextos.

## Conclusão

A validação da EDME-BR-VR alcançou os indicadores psicométricos e os níveis de confiabilidade satisfatórios, mostrando-se adequada para o uso. Esta versão reduzida preservou a estrutura original da escala, mantendo os quatro itens com maior carga fatorial por dimensão, totalizando 24 itens.

A análise fatorial exploratória da EDME-BR-VR, realizada a partir da invariância da idade, proporcionou uma compreensão mais aprofundada sobre os resultados e confirmou a validade do modelo reduzido. Portanto, ambas as escalas, tanto a versão completa, quanto a reduzida, demonstraram comportamento semelhante, proporcionando ao pesquisador a liberdade de escolher o instrumento mais adequado para suas investigações.

Porém, a EDME-BR-VR oferece vantagens práticas, pois é mais fácil de aplicar e compreender, além de exigir menos tempo de resposta dos participantes. Por fim, sugere-se a realização de novas pesquisas sobre o sofrimento moral, visando aprofundar o entendimento sobre as suas consequências e desenvolver as estratégias eficazes para seu manejo, com o intuito de fornecer suporte, tanto para os profissionais quanto para as organizações de saúde.

# Translation: Validación de la versión reducida de la Escala Brasileña de Distrés Moral en Enfermeros

**DOI:** 10.1590/1518-8345.7123.4454

**Authors:** Jordana Lopes Carvalho, Luis Felipe Dias Lopes, Flavia Regina Souza Ramos, Graziele de Lima Dalmolin

**Affiliations:** 1 Universidade Federal de Santa Maria, Santa Maria, RS, Brasil; 2 Universidade Federal de Santa Maria, Departamento de Ciências Administrativas, Santa Maria, RS, Brasil; 3 Universidade Federal de Santa Catarina, Florianópolis, SC, Brasil; 4 Universidade Federal de Santa Maria, Departamento de Enfermagem, Santa Maria, RS, Brasil

**Keywords:** Enfermería, Enfermeras y Enfermeros, Moral, Estrés Psicológico, Sistema Métrico, Estudio de Validación

## Abstract

validar un modelo de la versión reducida de la Escala Brasileña de Distrés Moral en Enfermeros.

estudio metodológico, con una muestra de 269 enfermeros de un hospital universitario público del sur de Brasil. Se utilizaron un cuestionario sociodemográfico y la Escala Brasileña de Distrés Moral en Enfermeros. Para el análisis estadístico descriptivo se emplearon el modelo de ecuaciones estructurales y el análisis de invarianza entre los grupos de edad.

en cuanto al perfil, predominaron los profesionales con edades entre 31 y 40 años, mujeres, casados, especialistas. El modelo estructural se ajustó a los datos, con índices de ajuste aceptables. El modelo propuesto presentó excelente consistencia interna, validez convergente y discriminante. El análisis del modelo estructural reveló que las cinco hipótesis fueron confirmadas y la invarianza entre grupos de edad indica que la forma en que se conceptualizan, miden e interrelacionan las variables en el modelo no afecta los resultados, independientemente de los grupos comparados

la escala alcanzó indicadores psicométricos satisfactorios, demostrando ser adecuada para su uso, manteniendo los elementos de validación relevantes por dimensión y ofreciendo ventajas prácticas. Se sugiere que se realicen más investigaciones para profundizar la comprensión de las consecuencias del distrés moral y desarrollar estrategias efectivas para su manejo.

## Destacados

**(1)** La versión reducida de la EDME-BR quedó compuesta por 24 preguntas y seis factores. **(2)** Para la validación se utilizaron el análisis de invarianza y el modelo de ecuaciones estructurales. **(3)** Los valores de consistencia interna y confiabilidad compuesta fueron adecuados. **(4)** Las pruebas indicaron suficiente relevancia predictiva del modelo propuesto. **(5)** La escala reducida propone un enfoque claro, accesible y con un menor tiempo de respuesta.

## Introducción

En el contexto de la práctica profesional de Enfermería, el enfermero necesita gestionar situaciones cotidianas que requieren decisiones ético-morales, dada la complejidad del sistema de salud. Estas decisiones abarcan aspectos organizativos, estructurales, relacionados con los equipos y los usuarios, y requieren posiciones y deliberaciones que suscitan en los profesionales una serie de emociones, como inquietud, malestar e incertidumbre ante los conflictos. Tales circunstancias pueden culminar en la experiencia de Distrés Moral (DM)^(1)^.

El DM es conceptualizado como la angustia interna asociada a conflictos externos entre valores, obligaciones y acciones, y que se manifiesta cuando el enfermero reconoce la línea de conducta adecuada a seguir, sin embargo, se ve impedido de actuar de acuerdo con su propio discernimiento y juicio^(1)^.

Así, las condiciones esenciales y suficientes para definir el DM están vinculadas a la conciliación de la experiencia moral y el desorden emocional, en una relación causal directa^(1)^. En el área de la salud, ante diferentes circunstancias éticamente frágiles, el DM se ha destacado como un fenómeno o experiencia de gran relevancia para la Enfermería, por la amplitud de causas y significados^(2-3)^.

Algunos estudios contemporáneos a nivel internacional indican niveles moderados de DM en enfermeras hospitalarias^(3-4)^. Estos hallazgos están en línea con los resultados de un estudio realizado con enfermeras brasileñas que actúan en el ambiente hospitalario, que reportó un nivel moderado de frecuencia e intensidad de DM^(5-6)^. Sin embargo, como agravante, el DM ha presentado implicaciones que resultan en disminución de la satisfacción laboral, desafíos éticos y aumento de la carga de trabajo, y que favorecen el aumento de la rotación profesional^(7)^, lo que exige una evaluación periódica de los equipos y de los ambientes de trabajo.

En este contexto, el avance de la investigación exige continuas revisiones y perfeccionamientos de los instrumentos de medición, que juegan un papel fundamental en la evaluación del sufrimiento moral entre los profesionales de la salud^(8)^. Actualmente, el DM es evaluado mediante la Escala Brasileña de Distrés Moral en Enfermeros (EDME-BR), que tiene en cuenta las peculiaridades de la profesión y de los contextos de prestación de servicios en Brasil^(9)^.

Este instrumento evidenció la presencia de DM con varios factores predictivos, señaló las similitudes entre las experiencias de los enfermeros en el contexto nacional e internacional, además de abordar el escenario de atención en diferentes niveles de complejidad y reconocer las particularidades del DM en Brasil^(9)^.

Al considerar lo anterior, se reafirma la importancia de medir la intensidad y la frecuencia del DM como un instrumento adecuado al ambiente laboral y escenario político-profesional, con foco en optimizar el tiempo de respuesta y facilitar la evaluación en diferentes contextos de desempeño. Esta necesidad se justifica por la particularidad de la EDME-BR, ya que se trata de una escala compuesta por 49 ítems, que fue utilizada en un estudio en el grupo de investigación con 269 enfermeros, cuando se reconoció el elevado tiempo de respuesta y numerosas negativas de participación debido a que es un instrumento extenso. Así, se visualizó la posibilidad, utilizando la base de datos del estudio, de realizar un nuevo análisis metodológico para reducir la escala. La relevancia de esto está asociada a que un mayor conocimiento sobre el DM, por medio de una escala de uso más fácil, puede contribuir significativamente a la comprensión que los trabajadores tienen sobre sus circunstancias y desafíos cotidianos.

Frente a esto, se planteó como pregunta de investigación: “La EDME-BR presenta validez y confiabilidad adecuadas en un modelo reducido?”. Para responderla, se estableció como objetivo validar un modelo de la versión reducida de la EDME-BR con profesionales de una institución hospitalaria pública de la Región Sur de Brasil.

De este modo, se presentaron como hipótesis para la reducción de la escala las posibles relaciones entre sus dimensiones, con la posterior contextualización:

H1: La percepción de Reconocimiento, Poder e Identidad Profesional (RPIP) se relaciona con la capacidad de Defensa de Valores y Derechos (DVD);

H2: El reconocimiento, poder e identidad profesional de los enfermeros están relacionados con la eficacia y cohesión de los Equipos de Trabajo (ET);

H3: Las Condiciones de Trabajo (CT) tienen un impacto directo en la percepción sobre los ET;

H4: Las CT (adversas) están relacionadas con la percepción y la ocurrencia de infracciones éticas en el ambiente de Enfermería.

H5: Existe una relación entre la capacidad de brindar Cuidado Seguro y Calificado (CSC) y la percepción de Infracciones Éticas (IEs);

H6: El análisis de invarianza entre los grupos de edad de los enfermeros supone que no existe diferencia entre los enfermeros de hasta 35 años y los de más de 35 años con relación a las cinco hipótesis probadas.

Finalmente, las hipótesis presentadas están de acuerdo con la literatura, dado que: la H1 sugiere que los enfermeros que se sienten más reconocidos y seguros en su identidad profesional están más inclinados a defender valores y derechos en el ambiente laboral^(10)^; la H2 sugiere que los enfermeros que perciben un mayor reconocimiento de sus competencias y valor profesional tienden a contribuir de manera más efectiva al trabajo en equipo, promoviendo un ambiente colaborativo y de apoyo mutuo^(11)^; la H3 sugiere que las mejores condiciones de trabajo promueven una mejor dinámica y cohesión del equipo^(12)^; la H4 sugiere que los entornos laborales con recursos insuficientes, alta carga de trabajo, presión intensa y soporte organizacional inadecuado aumentan el riesgo de compromiso ético, llevando a toma de decisiones que pueden no estar en conformidad con los estándares éticos y profesionales^(13)^; y, por último, la H5 indica que una mayor competencia y seguridad en el cuidado están asociadas a una menor percepción de infracciones éticas en el ambiente de trabajo^(14)^.

## Método

### Diseño del estudio

 Se trata de un estudio metodológico, enfocado en la validación de un instrumento a partir de modelación estadística, utilizando la base de datos del proyecto: “*Sofrimento moral em enfermeiros hospitalares: qual sua relação com clima ético e Burnout*?”, registrado en el Portal de Proyectos de la universidad de origen de la investigadora responsable. En el método de análisis mencionado, las pruebas de bondad de ajuste se relacionan con qué tan bien se ajusta un modelo a los datos. Estas pruebas ayudan a determinar si el modelo elegido reproduce las variables de respuesta para las que se optimizaron los parámetros o si es necesario considerar un modelo diferente ^(15)^.

### Escenario

El estudio se llevó a cabo en un hospital universitario público en la Región Sur de Brasil. Actualmente, la institución cuenta con 400 camas y actúa como referencia en la región por su tecnología y nivel de atención de mediana y alta complejidad, respaldada por un amplio equipo de trabajadores y estudiantes del área.

### Población, criterios de selección y definición de la muestra

Los participantes fueron 269 enfermeros que formaron parte del proyecto matricial citado anteriormente y que constituyeron la base de datos del mismo. El proyecto matricial tenía como población a 303 enfermeros que trabajaban en la institución en el momento de la recolección de datos; este valor se tomó como base para el uso de una fórmula para el cálculo de la muestra para la población finita, junto con un error muestral del 5% y un porcentaje estimado del 50%, lo que resultó en un mínimo de 171 participantes. Como criterio de inclusión, se seleccionaron a los enfermeros con al menos un mes de trabajo (tiempo mínimo estimado para la percepción sobre el ambiente laboral). Como criterio de exclusión, se enumeraron a los enfermeros que se encontraban de licencia o ausentes del trabajo durante el período de recolección de datos.

### Instrumentos utilizados para la recolección de la información y las variables del estudio

La base de datos del proyecto matricial se compuso de un cuestionario sociodemográfico y laboral, la escala EDME-BR, el Inventario de*Burnout* de Maslach ^(16)^ y el*Hospital Ethical Climate Survey* (HECS) ^(17)^. Sin embargo, para este estudio se utilizaron las variables del cuestionario sociodemográfico y laboral, así como la escala EDME-BR ^(9)^. El cuestionario sociodemográfico y laboral abordó las siguientes variables: género, estado civil, edad (transformada en dos grupos de edad, es decir, hasta 35 años y más de 35 años), formación, tiempo de formación, turnos de trabajo y carga horaria.

La EDME-BR ^(9)^es un instrumento construido y validado en Brasil en 2019, que resultó en una versión final de la escala con 49 ítems distribuidos en seis factores, para los cuales los valores de alfa de Cronbach variaron entre 0,91 y 0,96 ^(9)^. Estos son: Factor 1 - RPIP, con 11 preguntas (14, 15, 16, 34, 35, 36, 37, 38, 40, 41, 43); Factor 2 - CSC, con 11 preguntas (23, 24, 25, 26, 27, 28, 29, 30, 31, 32, 33); Factor 3 - DVD, con ocho preguntas (39, 42, 44, 45, 46, 47, 48, 49); Factor 4 - CT, con seis preguntas (8, 9, 10, 11, 12, 13); Factor 5 - IEs, con seis preguntas (17, 18, 19, 20, 21, 22); y Factor 6 - ET, con siete preguntas (1, 2, 3, 4, 5, 6, 7). Para cada ítem se aplica una escala Likert de siete puntos (desde 0 = ninguno/nunca hasta 6 = muy intenso/muy frecuente).

### Período y recolección de datos

El acceso a la base de datos se realizó en mayo de 2022. La recolección de datos del proyecto matricial se llevó a cabo entre abril y junio de 2019. Fue realizada por los autores y miembros del grupo de investigación que no eran cercanos a los participantes, y que fueron previamente capacitados y adquirieron experiencia en la recolección de datos cuantitativos.

Los enfermeros fueron invitados a participar en la investigación en sus lugares de trabajo, optando por responder el instrumento de inmediato o entregarlo posteriormente, después de firmar el Término de Consentimiento Libre e Informado (TCLE, en portugués), el cual fue firmado tanto por el participante como por el investigador, y se entregó una copia a cada uno.

Todos los enfermeros que aceptaron participar en la investigación recibieron información detallada sobre los objetivos y procedimientos, los posibles riesgos y beneficios involucrados, además de la garantía de su derecho a retirar su consentimiento para participar del estudio en cualquier momento, sin ninguna repercusión negativa ni exposición pública de su información.

En el caso de la entrega posterior de los instrumentos completados por los profesionales, se programó una búsqueda y se realizaron hasta tres intentos para recuperarlos, en fechas y momentos distintos. Además, durante el proceso de llenado, los recolectores garantizaron la libertad de los participantes al acercarse solamente al final, para recoger los instrumentos o para aclarar eventuales dudas.

Para garantizar la confidencialidad de la información, los formularios completados fueron almacenados por los recolectores en sobres sellados, que fueron entregados al investigador responsable.

### Procesamiento y análisis de los datos

La base de datos fue creada inicialmente en el programa*EpiInfo* ^®^(versión 6.4), a partir de una doble digitación independiente, con verificación de errores e inconsistencias. Las variables y escala seleccionadas en la base de datos fueron transportadas para su análisis en los*softwaresSmartPLS* ^®^(versión 4.1.0.1) y*Statistical Package for the Social Sciences*(SPSS^®^versión 26). Las variables sociodemográficas y laborales se analizaron mediante estadística descriptiva, con distribución de frecuencia absoluta (N) y relativa (%) para las variables categóricas.

Para validar la escala reducida, se utilizó la técnica del modelo de ecuaciones estructurales, mediante el método de mínimos cuadrados parciales, y el Análisis Multigrupo (MGA), más específicamente, el Análisis de Invarianza. El modelo elegido cumplió con el propósito de evaluar la estructura de interrelaciones entre las dimensiones de la escala propuesta y una ya validada, mediante la prueba de las hipótesis propuestas ^(18)^. Además, en el análisis de los datos se utilizó el modelo de ruta*Partial Least Squares - Structural Equation Modeling*(PLS-SEM) ^(16)^.

Este estudio adoptó el*Standardized Root Mean Square Residual*(SRMR), la distancia euclidiana al cuadrado (d_DEC_), la distancia geodésica (d_G_) y el*Normed Fit Index*(NFI) como criterios para evaluar el ajuste del PLS-SEM. Se adoptó 0,08 como valor umbral de SRMR ^(19)^y 0,8 como valor mínimo de NFI ^(20)^.

El modelo de medición de la EDME-BR presentó cinco hipótesis directas, que se conectan en tres dimensiones (dimensiones exógenas) con tres dimensiones endógenas (predictivas) que contienen 24 ítems (variables observadas) (Tabla 1). Tales hipótesis permiten evaluar la invarianza de la escala reducida entre grupos de edad, afirmando que la escala puede medir lo que pretende medir.

Asimismo, como parte integral de la evaluación PLS-SEM, se realizó el Análisis Factorial Confirmatorio (AFC) que compara los grupos de edad, con miras a confirmar y perfeccionar los ítems y dimensiones dispuestos en el modelo. La consistencia interna entre los componentes se verificó, en cada dimensión, mediante el Alfa de Cronbach (AC). La validez discriminante se estableció mediante los criterios de Fornell-Larcker (FL) y*Heterotrait-Monotrait Ratio* (HTMT), para determinar en qué medida una dimensión dentro de sus componentes difería de otra ^(21)^.

La raíz cuadrada del valor de la Varianza Media Extraída (VME) (Tabla 3) de cada dimensión fue calculada y comparada con los valores de la Matriz de Correlación y del criterio HTMT, utilizando el procedimiento de*bootstrap*, el cual tuvo valores menores que uno para el límite superior (97,5% de confianza).

También en esta etapa se verificaron la confiabilidad de consistencia interna, la validez convergente y discriminante^(20)^, es decir, se contempló la asunción de la Varianza Media Extraída (AVE > 0,5), el Alfa de Cronbach (0,7 < AC < 0,95) y la confiabilidad compuesta (0,7 < CC < 0,95) para el modelo^(22-23^. Y, para evaluar si el modelo estructural representó las teorías subyacentes al modelo de medición, se permitió analizar la capacidad predictiva del modelo y las relaciones entre las dimensiones de este modelo, y se consideró el análisis de (multi)colinealidad estimando el*Variance Inflation Factor*(VIF), que tuvo valores para las variables latentes inferiores a 5^(22)^.

Para evaluar la significancia de los valores de los coeficientes de ruta en las relaciones directas entre los factores, con base en el valor*t* de*Student*, se utilizó la técnica de*bootstrapping* con 5.000 submuestras. La relación directa entre los factores se midió a partir de coeficientes estructurales (β’s), y la relación de ruta se consideró significativa, al obtener niveles de significancia del 5%, con un valor*t* inferior a 1,96^(18)^.

En cuanto al procedimiento de reducción, se utilizaron técnicas de evaluación del modelo estructural, con el fin de verificar su consistencia interna y validez convergente. En la evaluación de la validez discriminante del modelo, se optó por considerar las cargas factoriales (λ) más expresivas para cada una de las dimensiones. A continuación, el modelo fue sometido a una evaluación de su estructura, incluyendo el análisis de la precisión (R²) y de la relevancia predictiva (Q²)^(18^.

Una etapa crucial en la evaluación de un modelo PLS-SEM es la prueba de su precisión y relevancia predictiva. La precisión predictiva se evaluó mediante el coeficiente de determinación (R²) y el respectivo valor de p, que proporciona el grado de varianza explicado en cada dimensión endógena^(18)^. El valor de R² varía entre 0 y 1; cuanto mayor sea su valor, mayor será la precisión predictiva^(18)^. Además, se evaluó la significancia de R², con valores que indican diferentes niveles de precisión predictiva: 0,075 < R² ≤ 0,19 (efecto moderado) y R² > 0,19 (efecto fuerte)^(22-23)^.

La relevancia predictiva se evaluó mediante el procedimiento de los ojos vendados, que calculó el valor de Q² utilizando la técnica de*blindfolding*. Un valor de Q² superior a 0 se considera indicativo de relevancia predictiva^(18-23)^. Los valores de Q² varían entre 0,075 y 0,25 para un grado moderado de relevancia predictiva, mientras que Q² > 0,25 es indicativo de un grado fuerte de relevancia predictiva^(23-24)^.

### Aspectos éticos

Todos los preceptos éticos de la investigación con seres humanos fueron respetados, de acuerdo con la Resolución nº 466/12, con el proyecto aprobado por Comité de Ética en Investigación (CEI) local bajo Dictamen nº 2.764.702.

## Resultados

### Perfil de los participantes

Participaron de la investigación 269 enfermeros, de los cuales 83 (30,9%) tenían hasta 35 años y 186 (69,1%) tenían más de 35 años. La mayoría de los participantes eran mujeres (88,1%) y el 75,09% estaban casados. En cuanto a la formación académica, el 54,37% de los participantes tenía una especialización y el 53,17% había completado su formación entre seis y 15 años antes de la investigación. Con relación a la carga horaria de trabajo, la mayoría de los enfermeros (65,06%) trabajaba 30 horas o más por semana, y el 39,04% tenía turnos de trabajo mixtos. Cabe destacar que, al considerar que el tiempo de trabajo en el hospital fue un criterio de inclusión, la mediana de tiempo de trabajo en la institución fue de cuatro (AIq10) años, 187 (69,5%) tenían cuatro años o más, y solamente cuatro (1,49%) habían trabajado durante un mes, mientras que todos los demás tenían más de seis meses de experiencia.

### Pruebas de ajuste del modelo

Los resultados confirmaron que el modelo estructural sugerido se ajustaba a los datos después de ocho interacciones con índices aceptables, con SRMR = 0,062, d_DEC_= 2,563, d_G_= 1,605 y NFI = 0,848.

### Evaluación del modelo de medición

En esta etapa se evaluaron los tres criterios: confiabilidad de consistencia interna, validez convergente y discriminante, considerando la varianza media extraída.

La consistencia interna entre los componentes, en cada dimensión, se verificó mediante el AC y la CC. Los valores de AC de todas las dimensiones variaron entre 0,798 y 0,930. Además, los valores de Confiabilidad Compuesta (CC) oscilaron entre 0,817 y 0,947. Finalmente, los VME presentaron valores entre 0,701 y 0,950. En cuanto a las cargas factoriales, para la escala EDME-BR-R los valores de cargas factoriales fueron superiores a 0,6 (Figura 2), por lo que estos resultados afirmaron la garantía de consistencia interna entre los indicadores y sus respectivos factores. La Tabla 1presenta los valores de la evaluación de todas las dimensiones del modelo de medición.

Al evaluar la validez discriminante del modelo, se utilizaron FL y HTMT como criterios principales. El criterio FL comparó la raíz cuadrada del valor de la VME de cada dimensión medida con los valores de la matriz de correlación. Se observó que la menor VME (0,802) es superior a la mayor correlación CT vs. CSC (0,679), por lo que se estableció el criterio. Por su parte, el criterio HTMT determinó que los límites superiores estimados fueron inferiores a 1.0. Así, se verificó que el modelo presenta validez discriminante y, de este modo, cumple con los requisitos.

En resumen, las evaluaciones del modelo de medición en cuanto a consistencia interna, validez convergente y discriminante cumplieron con sus requisitos, validando empíricamente la adecuación del modelo de medición en este estudio (Tablas 1 y 2).

**Tabla 1 -:** Evaluación del modelo de medición – consistencia interna y validez convergente. Santa Maria, RS, Brasil, 2019

EDME-BR* (Dimensiones)	**AC** ^†^		**CC** ^‡^		**VME** ^§^	
	**≤ 35** ^ǁ^	**> 35** ^¶^	**≤ 35** ^ǁ^	**> 35** ^¶^	**≤ 35** ^ǁ^	**> 35** ^¶^
RPIP**	0,857	0,798	0,904	0,817	0,701	0,865
DVD^††^	0,875	0,883	0,912	0,904	0,721	0,919
IEs^§§^	0,898	0,930	0,929	0,936	0,766	0,950
CSC^‖‖‖^	0,925	0,899	0,947	0,900	0,816	0,930
CTs^¶¶^	0,868	0,825	0,909	0,835	0,714	0,882
ETs***	0,785	0,830	0,861	0,838	0,608	0,887

*EDME-BR = Escala Brasileña de Distrés Moral en Enfermeros;^†^AC = Alfa de Cronbach;^‡^CC = Confiabilidad Compuesta;^§^VME = Varianza Media Extraída;^ǁ^≤ 35 = Menor e igual a 35 años;^¶^> 35 = Más de 35 años; **RPIP = Reconocimiento, Poder e Identidad Profesional;^††^DVD = Defensa de Valores y Derechos;^§§^IEs = Infracciones Éticas;^ǁǁ^CSC = Cuidado Seguro y Calificado;^¶¶^CTs = Condiciones de Trabajo; ***ETs = Equipos de Trabajo

**Tabla 2 -:** Criterio de Fornell-Larcker y HTMT* del modelo factorial. Santa Maria, RS, Brasil, 2019

**Dim.** ^†^	^‡^	**Matriz de Correlación de Pearson**					
		**RPIP** ^§^	**DVD** ^‖‖^	**IEs** ^¶^	**CSC** ^**^	**CTs** ^††^	**ETs** ^‡‡^
RPIP^§^	0,821	1,000					
DVD^‖‖^	0,885	0,632	1,000				
IEs^¶^	0,856	0,476	0,607	1,000			
CSC**	0,805	0,586	0,527	0,561	1,000		
CTs^††^	0,897	0,555	0,664	0,674	0,679	1,000	
ETs^‡‡^	0,802	0,551	0,576	0,575	0,665	0,647	1,000
		L**í**mite Superior (HTMT*)_97,5%_					
DVD^‖‖^		0,733					
IEs^¶^		0,811	0,816				
CSC**		0,742	0,758	0,790			
CTs^††^		0,740	0,648	0,707	0,821		
ETs^‡‡^		0,866	0,738	0,873	0,709	0,785	

*HTMT =*Heterotrait-Monotrait Ratio*;^†^Dim. = Dimensión;^‡^= Raíz de la Varianza Media Extraída;^§^RPIP = Reconocimiento, Poder e Identidad Profesional;^‖‖^DVD = Defensa de Valores y Derechos;^¶^IEs = Infracciones Éticas; **CSC = Cuidado Seguro y Calificado;^††^CTs = Condiciones de Trabajo;^‡‡^ETs = Equipos de Trabajo

### Análisis del modelo estructural

Tras analizar la colinealidad entre las dimensiones y calcularse los valores del VIF entre 1,000 < VIF < 1,664, el modelo no mostró fuertes correlaciones. Por lo tanto, los valores de los coeficientes de regresión (β) fueron estimados utilizando la técnica de*bootstrapping*para 5.000 submuestras. Los valores y sus significancias se describen en la Tabla 3.

**Tabla 3 -:** Resultados de efectos directos entre dimensiones. Santa Maria, RS, Brasil, 2019

**Relación de ruta (Hipótesis)**		**Coeficientes de ruta**		**Desviación estándar**	Estad. t.*	**p-valor** ^†^	**Resultado**
H1^‡^	RPIP^§^→ DVD^‖‖^	0,575	0,040	14,526	0,000	Soporta
H2^¶^	RPIP^§^→ ETs**	0,492	0,056	8,763	0,000	Soporta
H3^††^	CTs^‡‡^→ ET**	0,315	0,059	5,288	0,000	Soporta
H4^§§^	CT^‡‡^→ IEs^‖‖‖‖^	0,226	0,062	3,652	0,000	Soporta
H5^¶¶^	CSC*** → CT^‡‡^	-0,521	0,056	9,349	0,000	Soporta

*Estad. t. = Prueba*t*de Student;^†^p-valor = Significancia;^‡^H1 = Hipótesis 1;^§^RPIP = Reconocimiento, Poder e Identidad Profesional;^ǁ^DVD = Defensa de Valores y Derechos;^¶^H2 = Hipótesis 2; **ETs = Equipos de Trabajo;^††^H3 = Hipótesis 3;^‡‡^CTs = Condiciones de Trabajo;^§§^H4 = Hipótesis 4;^ǁǁ^IEs = Infracciones Éticas;^¶¶^H5 = Hipótesis 5; ***CSC = Cuidado Seguro y Calificado

Todas las hipótesis propuestas confirmaron relaciones significativas (p < 0,05) entre las dimensiones; por lo tanto, las hipótesis fueron empíricamente respaldadas a partir de los valores de*t* para un nivel de significancia del 5%. Se observó que el cuidado seguro y calificado se relaciona negativamente con las infracciones éticas; diez relaciones fueron significativas (Figura 1).

El análisis (Figura 1) reveló que el modelo mostró dimensiones con coeficientes de determinación (R²) que indican grados fuertes de explicación (R² > 0,19) para las variables predictivas, lo que confirma que estos resultados fueron consistentes. Además, los valores de relevancia predictiva de las dimensiones endógenas, estimados mediante el método de*blindfolding*, mostraron valores de Q² superiores a 0, lo que indica una buena capacidad de predicción del modelo. Más específicamente, los valores de Q² variaron entre 0,323 y 0,498.

A continuación, en la Tabla 4, se presenta el análisis de invarianza del modelo, referente a la hipótesis H6, donde se comparan las hipótesis H1 a H5 entre los grupos de edad de hasta 35 años y de más de 35 años.

**Figura 1 -:** Modelo de ecuaciones estructurales final. Santa Maria, RS, Brasil, 2019

*RPIP = Reconocimiento, Poder e Identidad Profesional;^†^DVD = Defensa de Valores y Derechos;^‡^CTs = Condiciones de Trabajo;^§^ETs = Equipos de Trabajo;^ǁ^CSC = Cuidado Seguro y Calificado;^¶^IEs = Infracciones Éticas

**Tabla 4 -:** Análisis multigrupo (MGA*) mediante pruebas de Henseler y Permutación. Santa Maria, RS, Brasil, 2019

**Hip.** ^‡^	**Relación**	**Coeficientes Estructurales**			**p-valor** ^†^ **(diferencias)**		**Resultado**
		**Ha.35** ^§^	**Ma.35** ^ǁ^	**Ha-Ma** ^¶^	*Henseler’s* MGA**	**Prueba de Permutación**	
H1^††^	RPIP^‡‡^→ DVD^§§^	0,640	0,549	0,090	0,283	0,306	No / No
H2^ǁǁ^	RPIP^‡‡^→ ETs^¶¶^	0,426	0,516	-0,091	0,429	0,469	No / No
H3***	CTs^†††^→ ETs^¶¶^	0,371	0,312	0,059	0,625	0,642	No / No
H4^‡‡‡^	CTs^†††^→ IEs^§§§^	0,189	0,245	-0,057	0,679	0,688	No / No
H5^ǁǁǁ^	CSC^¶¶¶^→ CTs^†††^	-0,620	-0,477	-0,143	0,245	0,222	No / No

*MGA =*Multi Group Analysis*;^†^p-valor = Significancia;^‡^Hipótesis;^§^Ha. 35 = Hasta 35 años;^ǁ^Ma. 35 = Más de 35 años;^¶^Ha – Ma = Diferencia entre hasta y más de 35 años;^††^H1 = Hipótesis 1;^‡‡^RPIP = Reconocimiento, Poder e Identidad Profesional;^§§^DVD = Defensa de Valores y Derechos;^ǁǁ^H2 = Hipótesis 2;^¶¶^ETs = Equipos de Trabajo; ***H3 = Hipótesis 3;^†††^CTs = Condiciones de Trabajo;^‡‡‡^H4 = Hipótesis 4;^§§§^IEs = Infracciones Éticas;^ǁǁǁ^H5 = Hipótesis 5;^¶¶¶^CSC = Cuidado Seguro y Calificado

Se observa, en laTabla 4, que tanto para las pruebas de*Henseler*como para la prueba de permutación, no existe diferencia significativa entre los coeficientes de los diferentes grupos de edad (p > 0,05). Por lo tanto, se concluye que el modelo propuesto no presenta problemas de invarianza y, así, la escala se comporta consistentemente entre los grupos^(25)^.

El alfa de Cronbach para el modelo EDME-BR mostró buena confiabilidad en todos sus factores (0,95), valor inferior al de la versión original (0,98). La versión reducida de la EDME-BR, que contiene 24 ítems, fue obtenida a partir del cálculo de índices de confiabilidad (AC y CC), considerando, como criterio de selección de indicadores, los cuatro ítems por dimensión con mayor carga factorial.

Asimismo, para preservar las características de la escala, los cambios realizados no modificaron las instrucciones ni la escala de respuestas, ni interfirieron en la calidad de los resultados obtenidos. Por lo tanto, la EDME-BR, en su versión reducida, quedó compuesta por 24 preguntas que evalúan los siguientes factores:

Factor 1 - Reconocimiento, Poder e Identidad Profesional (RPIP), con cuatro preguntas: 14) Sentirse discriminado con relación a otros profesionales; 36) Trabajar bajo presión debido a la insuficiencia de tiempo para alcanzar metas o realizar tareas; 40) Sentirse irrespetado por superiores jerárquicos; y 41) Reconocer actitudes éticamente incorrectas de los gestores o superiores jerárquicos.

Factor 2 - Cuidado Seguro y Calificado (CSC), con cuatro preguntas: 24) Reconocer que la contención del usuario es inadecuada; 25) Reconocer que las demandas de continuidad del cuidado del paciente/usuario no son atendidas; 26) Reconocer la falta de resolutividad de las acciones de salud debido a los problemas sociales; y 28) Reconocer que las acciones educativas con el usuario son insuficientes.

Factor 3 - Defensa de Valores y Derechos (DVD), con cuatro preguntas: 39) Vivenciar conductas asistenciales que desconsideran las creencias y la cultura de los pacientes; 42) Sentirse presionado a aceptar o guardar silencio ante fraudes en beneficio de la institución; 45) Reconocer situaciones de falta de respeto/maltratos por parte de los profesionales hacia el usuario; y 46) Reconocer situaciones de falta de respeto al derecho a la privacidad/intimidad del usuario.

Factor 4 - Condiciones de Trabajo (CTs), con cuatro preguntas: 8) Reconocer que los materiales de consumo son insuficientes; 10) Reconocer que los equipos/materiales permanentes disponibles son insuficientes; 12) Reconocer que la estructura física del servicio es insuficiente; y 13) Reconocer que la estructura física del servicio es inadecuada.

Factor 5 - Infracciones Éticas (IEs), con cuatro preguntas: 17) Vivenciar omisión por parte del médico; 18) Vivenciar imprudencia por parte del médico; 19) Vivenciar omisión por parte del enfermero; y 20) Vivenciar imprudencia por parte del enfermero.

Factor 6 - Equipos de Trabajo (ETs), con cuatro preguntas: 1) Trabajar con un número insuficiente de profesionales para satisfacer la demanda; 3) Experimentar condiciones de sobrecarga de trabajo; 5) Trabajar con enfermeros sin la preparación adecuada; y 6) Trabajar con auxiliares y técnicos de enfermería sin la preparación adecuada.

Para cada ítem, se aplica una escala Likert de siete puntos (desde 0 = ninguno/nunca hasta 6 = muy intenso/muy frecuente).

## Discusión

Con base en los resultados, fue posible validar la EDME-BR en su versión reducida, denominándose “Escala Brasileña de Distrés Moral en Enfermeros – Versión reducida” (EDME-BR-VR), que presentó una reducción de 25 preguntas, en comparación a la versión original. La EDME-BR-VR se mantuvo con 24 preguntas, conservando los factores, es decir: Reconocimiento, Poder e Identidad Profesional; Cuidado Seguro y Calificado; Defensa de Valores y Derechos; Condiciones de Trabajo; Infracciones Éticas; y Equipos de Trabajo. Sin embargo, ahora, cada uno está compuesto por solamente cuatro ítems.

En este modelo el estudio obtuvo SRMR = 0,062 y NFI = 0,848. La raíz cuadrada media de los residuos estandarizados es una medida de ajuste que tiene como objetivo evaluar el grado de adecuación del modelo, y debe ser inferior a 0,08 para un resultado satisfactorio^(19-20)^. El NFI es la razón entre el χ² del modelo independiente y el χ² del modelo que fue probado; no penaliza la complejidad del modelo y es sensible al tamaño de la muestra. Es una comparación entre la matriz de covarianza prevista por el AFC y la matriz original de lo que es previsto por el modelo, debiendo ser superior a 0,8^(19-20)^.

Respecto a las hipótesis presentadas y verificadas, la relación entre RPIP y DVD puede explicarse por el concepto de “*empowerment*” profesional, que implica tener el poder, autoridad y capacidad para ejecutar acciones basadas en valores éticos y profesionales. Los enfermeros que perciben un mayor “*empowerment*” en el trabajo se sienten más capacitados para actuar de acuerdo con sus valores profesionales, que incluyen defender los derechos de los pacientes y adherirse a estándares éticos elevados^(26-27)^.

Una fuerte identidad profesional y el reconocimiento en el ambiente de trabajo contribuyen a una mejor dinámica de equipo. La claridad del rol y el reconocimiento de las habilidades de los enfermeros se asocian con una mejor comunicación y colaboración entre los miembros del equipo, lo que facilita la cohesión y la eficacia del grupo^(28)^.

La influencia de las condiciones de trabajo en la percepción de los equipos de enfermería puede entenderse por medio de la teoría de los recursos de trabajo, que postula que los ambientes de trabajo con recursos funcionales mejoran la satisfacción y el compromiso de los empleados, además de promover relaciones positivas entre los colegas. Las mejores condiciones de trabajo están asociadas a una mejor calidad de cuidados y a la satisfacción laboral entre los enfermeros^(29)^.

Se sabe que las condiciones de trabajo adversas pueden crear dilemas éticos, donde los enfermeros se ven obligados a tomar decisiones que comprometen los estándares éticos. Ambientes estresantes y con recursos insuficientes aumentan los conflictos éticos que enfrentan los enfermeros, evidenciando la relación entre las condiciones de trabajo y la ocurrencia de infracciones éticas^(30)^.

La capacidad de brindar un cuidado seguro y calificado puede verse comprometida en entornos donde prevalecen infracciones éticas, creando una relación negativa entre estas dimensiones. En otras palabras, los impactos resultantes del DM pueden comprometer el desempeño de los enfermeros e influir negativamente en su eficacia para cumplir los objetivos de salud establecidos^(13)^.

El hecho de que todas estas hipótesis no difieran entre los grupos de edad de hasta 35 años y de más de 35 años sugiere una universalidad en las percepciones y experiencias de los enfermeros, independientemente de la edad. Esto puede indicar que las estructuras profesionales, éticas y organizacionales se perciben de manera consistente a lo largo de la carrera de Enfermería, reforzando la importancia de abordar estas dimensiones en las estrategias de desarrollo profesional y políticas de salud.

Un estudio realizado en el contexto internacional con profesionales de Enfermería destacó niveles significativos en la intensidad y frecuencia del sufrimiento moral por condiciones laborales insuficientes^(31)^.

Las hipótesis de realización profesional y DM no mostraron una relación significativa entre sus puntajes. Esto concuerda con los hallazgos de un estudio realizado con 280 enfermeros, que examinó la relación entre la satisfacción laboral y el sufrimiento moral. La satisfacción es un constructo inverso al DM, es decir, a mayor grado de DM, menor será la realización profesional^(32)^.

Los puntajes de sufrimiento moral y satisfacción profesional mostraron una correlación negativa entre sus variables, indicando que estos constructos son inversamente proporcionales^(33)^. Los niveles de realización profesional pueden estar asociados a aspectos organizacionales, de interacciones, de autonomía y de remuneración que, en niveles bajos, pueden provocar manifestaciones físicas, debido a la sobrecarga de trabajo, o emocionales, como el estrés y la ansiedad, generadores frecuentes de la insatisfacción laboral y del sufrimiento moral^(7)^.

Los sentimientos de desvalorización e impotencia por parte de los profesionales pueden ocasionar conflictos éticos/morales, llevando a la insatisfacción en el trabajo^(33)^. Sin embargo, se pudo constatar, en la versión reducida de la escala, que los mayores promedios de DM están vinculados a los constructos cuidado seguro y calificado, condiciones de trabajo, seguidos de infracciones éticas.

Estos resultados convergen con otros estudios realizados con la aplicación de la escala original. Un estudio con enfermeros hospitalarios demostró los mayores índices de sufrimiento moral relacionados con los constructos “cuidado seguro y calificado”, “condiciones de trabajo” y “equipos de trabajo”^(34)^.

Además, de los 269 enfermeros participantes, en su mayoría mujeres, se obtuvo una prevalencia de edad entre 31 y 40 años. Un estudio que investigó el DM en enfermeros obtuvo resultados similares, con un perfil representado principalmente por mujeres, con una edad promedio de 37 años^(35)^.

La aparición de DM está asociada a la naturaleza subjetiva del trabajo, la experiencia profesional y las condiciones organizacionales, especialmente con los desafíos que se enfrentan en el trabajo diario^(8)^. Un evento desencadenante de DM, ya sea frecuente o esporádico, puede aportar datos importantes que deben ser analizados, con énfasis en la relevancia de las circunstancias que lo rodean.

Aunque la literatura ha sugerido diversas definiciones de DM, hay un consenso de que este surge de limitaciones inadecuadas en la práctica clínica que impactan la autonomía moral de los profesionales de la salud. Por lo tanto, para mitigar el sufrimiento moral, es crucial que los instrumentos de medición proporcionen información relevante sobre estas limitaciones^(36)^, siendo la EDME-BR-VR una opción válida y confiable.

Al comparar los resultados obtenidos en la EDME-BR y en la EDME-BR-VR, se pudo constatar que tanto la escala en su versión completa (49 ítems) como en su versión reducida (24 ítems) presentaron buena confiabilidad y validez convergente. Los coeficientes de explicación presentaron grados moderados para los factores: Condiciones de Trabajo, Defensa de Valores y Derechos, Interacciones Éticas y Cuidado Seguro y Calificado.

En general, el estudio contribuyó a resaltar la importancia de evaluar el DM en enfermeros, ya que tiene impacto directo en la salud mental y en su trabajo cotidiano, interfiriendo en sus acciones y relaciones, y en la calidad del cuidado al paciente. Así, la escala reducida EDME-BR facilita un abordaje claro, accesible, con menor tiempo de respuesta, demostrando confiabilidad en todos los ítems evaluados, lo que la convierte en un instrumento válido para ser utilizado en la evaluación de DM en enfermeros.

Los factores de reconocimiento, poder e identidad profesional mostraron grados fuertes, lo que implica una moderada capacidad para producir predicciones precisas y replicabilidad en nuevos datos.

Las limitaciones de este estudio se deben a que se realizó solamente en una institución hospitalaria, lo que imposibilita las comparaciones con otros contextos.

## Conclusión

La validación de la EDME-BR-VR alcanzó indicadores psicométricos y niveles de confiabilidad satisfactorios, demostrando ser adecuada para su uso. Esta versión reducida preservó la estructura original de la escala, manteniendo los cuatro ítems con mayor carga factorial por dimensión, totalizando 24 ítems.

El análisis factorial exploratorio de la EDME-BR-VR, realizado a partir de la invarianza de la edad, proporcionó una comprensión más profunda sobre los resultados y confirmó la validez del modelo reducido. Por lo tanto, ambas escalas, tanto la versión completa como la reducida, demostraron un comportamiento similar, brindando al investigador la libertad de elegir el instrumento más adecuado para sus investigaciones.

Sin embargo, la EDME-BR-VR ofrece ventajas prácticas, ya que es más fácil de aplicar y comprender, además de requerir menos tiempo de respuesta por parte de los participantes. Finalmente, se sugiere realizar nuevas investigaciones sobre el sufrimiento moral, con el objetivo de profundizar la comprensión de sus consecuencias y desarrollar estrategias efectivas para su manejo, y así brindar apoyo tanto a los profesionales como a las organizaciones de salud.
